# Self-reported chronic kidney disease and the risk of all-cause and cause-specific mortality: outcome-wide association study of 54 causes of death in the National Health Interview Survey

**DOI:** 10.1186/s12882-022-02771-1

**Published:** 2022-04-30

**Authors:** Dagfinn Aune, Xibin Sun, Jing Nie, Wentao Huang, Bing Liao, Yafeng Wang

**Affiliations:** 1 Department of Epidemiology and Biostatistics, School of Public Health, Imperial College London, St. Mary’s Campus, Norfolk Place, Paddington, London, W2 1PG UK; 2 Department of Nutrition, Oslo New University College, Oslo, Norway; 3 Department of Endocrinology, Morbid Obesity and Preventive Medicine, Oslo University Hospital, Oslo, Norway; 4 Unit of Cardiovascular and Nutritional Epidemiology, Institute of Environmental Medicine, Karolinska Institutet, Stockholm, Sweden; 5 School of Public Health, Guangdong Pharmaceutical University, Guangzhou, China; 6 Department of Sociology & Institute for Empirical Social Science Research, School of Humanities and Social Sciences, Xi’an Jiatong University, Xi’an, China; 7 School of Nursing, Guangdong Pharmaceutical University, Guangzhou, China; 8 Department of Epidemiology and Biostatistics, School of Health Sciences, Wuhan University, 185 Donghu Road, Wuchang District, Wuhan, 430071 China

**Keywords:** Chronic kidney disease, Blood pressure, Mortality, Prospective, National Health Interview Survey

## Abstract

**Background:**

A diagnosis of chronic kidney disease has been strongly associated with cardiovascular disease and mortality in a number of studies, but the association with specific causes of death has not been assessed in detail. We analysed the association between chronic kidney disease and all-cause mortality and 54 causes of death in the National Health Interview Survey, a prospective study of 210,748 US adults.

**Methods:**

We used multivariable Cox regression models to estimate hazard ratios (HRs) and 95% confidence intervals (CIs) for all-cause and cause-specific mortality associated with self-reported chronic kidney disease. Men and women aged 18–84 years were recruited between 1997 and 2004 and followed up for mortality through December 31, 2006.

**Results:**

During an average of 6 years follow-up, 9564 deaths occurred. A history of chronic kidney disease vs. no chronic kidney disease was associated with increased risk of all-cause mortality (HR = 2.69, 95% CI: 2.38–3.04), and mortality from septicemia (5.65, 2.84–11.25), viral hepatitis (10.67, 2.43–46.95), other infectious parasitic diseases (10.58, 3.59–31.21), total cancer (1.48, 1.05–2.09), lung cancer (1.94, 1.10–3.44), kidney cancer (4.74, 1.81–12.41), diabetes mellitus (8.57, 5.60–13.11), circulatory disease overall (3.36, 2.70–4.18) and 11 specific circulatory diseases with the strongest associations observed for primary hypertension/renal disease (13.60, 6.42–28.84), hypertensive heart/renal disease (10.72, 2.47–46.49), and other diseases of circulatory system (7.36, 3.22–16.81). Elevated risk was also observed for alcoholic liver disease (5.63, 1.90–16.66), other chronic liver disease (4.41, 1.74–11.17), kidney failure (13.07, 8.23–20.77), and five other causes of death.

**Conclusions:**

A history of chronic kidney disease was associated with increased risk of all-cause mortality and 27 out of 54 causes of death. Further studies are needed to clarify associations with less common causes of death.

**Supplementary Information:**

The online version contains supplementary material available at 10.1186/s12882-022-02771-1.

## Introduction

Chronic kidney disease is an important cause of morbidity and mortality and accounted for 1.23 million deaths, 28.5 million years of life lost (YLLs) and 35.8 million disability-adjusted life years (DALYs) globally in 2017 according to the Global Burden of Disease Project [1, 2]. A total of nearly 700 million prevalent cases were recorded globally in 2017, giving an overall prevalence of 9.1% [3]. Persons with chronic kidney disease have reported a lower quality of life, and are at increased risk of cardiovascular disease and premature mortality [4, 5]. Major risk factors for chronic kidney disease include diabetes [6], high blood pressure [6], and cardiovascular disease [7]. In addition, some studies have suggested lifestyle factors such as adiposity [8], diet [9] and physical activity [10] may be important.

Many studies have investigated the association between chronic kidney disease and cardiovascular disease and mortality, and are consistent in showing increased risk for cardiovascular disease and all-cause mortality [4, 5, 11–19]. However, relatively few studies have investigated the association between chronic kidney disease and very detailed causes of death [20]. In the GLOMMS-1 study from the UK, strong increases in risk was observed across a number of causes of death including ischemic heart disease, cerebrovascular disease, other forms of heart disease, diseases of arteries, arterioles and capillaries, influenza and pneumonia, chronic lower respiratory diseases, malignant digestive organ cancers, diabetes mellitus, mental disorders, and renal failure [20], and another study from Taiwan reported similar results [21]. However, we are not aware of other studies that have looked at more detailed causes of death in chronic kidney disease  patients. To provide a more detailed assessment across causes of death, we investigated the association between self-reported chronic kidney disease and risk of all-cause mortality as well as 54 causes of death in the National Health Interview Survey.

## Methods

### Study population

The National Health Interview Survey (NHIS) is an ongoing national cross-sectional survey, conducted annually by the National Center for Health Statistics in collaboration with the US Census Bureau since 1957, and used a multistage sample design to monitor the health of the US civilian non-institutionalized population, as previously described [22]. A total of 237,847 participants who were 18–84 years of age from 8 waves conducted during 1997 to 2004 (linked to mortality data through December 2006) participated in the study. We excluded 27,099 respondents with history of coronary heart disease, stroke or cancer at baseline or missing data on kidney disease, leaving 210,748 participants for inclusion in the final analysis sample. All data were based on self-reports and obtained via household roster section of the questionnaire of participants. The design of the NHIS has been reviewed and approved by Institutional Review Board at the Centers for Disease Control and Prevention. Written informed consent was obtained from all subjects.

### Mortality

This analysis included all-cause and cause-specific mortality as outcomes. The 10th revision of the International Statistical Classification of Diseases, Injuries, and Causes of Death (ICD-10) was used to classify cause-specific deaths (Supplementary Table 1). Linkages were made to the National Death Index through 2006 to identify deaths that occurred during the follow-up.

### Assessment of chronic kidney disease

The baseline questionnaire included the question “during the past 12 months, have you been told by a doctor or other health professional that you had weak or failing kidneys? Do not include kidney stones, bladder infections or incontinence”, which was used to assess self-reported chronic kidney disease status.

### Covariates

Covariates were selected a priori based on previous literature and availability in the dataset. Baseline characteristics associated with chronic kidney disease and mortality were included as covariates from the survey and included demographic variables such as age, sex, race (Hispanic, non-Hispanic White, non-Hispanic Black, and Other), education level (less than high school degree, high school degree, more than high school degree) and income level (low, middle, high) and lifestyle factors including BMI (< 25, 25- < 30, ≥30 kg/m^2^), leisure-time physical activity (inactive, insufficient, sufficient), smoking status (never, former, and current cigarette smokers), alcohol intake (lifetime abstainer, former drinker, current drinker), similar to our previous analysis [22]. Based on the family income, the poverty ratio was calculated, which reflects the annual family income relative to the federal poverty level (PIR). A score of 1 represents the poverty level, a score below 1 represents incomes below the poverty level, and a score above 1 represents incomes above the poverty level. The household income was divided into low (PIR, ≤1), moderate (PIR, 1 to < 4), and high (PIR, ≥ 4) groups. For covariates with missing information we created a separate missing data category. The amount of missing data was low for these covariates, ranging from 0.5% to 3.4%.  

### Statistical analysis

Analyses accounted for the complex survey design employed in NHIS by utilizing sample weights, primary sampling and clustering units via the Taylor series (linearization) method. Person-years of follow-up were calculated for each participant from the recruitment date to the date of death or end of the study period (31 December 2006). Hazard ratios (HRs) and 95% confidence intervals (CIs) for the association between chronic kidney disease and all-cause and cause-specific mortality were calculated using multivariable Cox proportional hazards regression models with adjustment for age, sex, race, education, income, BMI, leisure-time physical activity, alcohol, smoking status and survey year. The proportional hazards assumption was tested in relation to all-cause mortality, using the log-log plot of survival [23]. Analyses stratified by age, sex, ethnicity/race, alcohol, smoking status, and BMI were conducted to test for interactions and to better be able to rule out residual confounding by these risk factors. Tests for interactions were made by creating cross-product terms between chronic kidney disease and each variable and adding this to the model. The significance of the interactions were tested using a likelihood ratio test. E-values were calculated to estimate the strength of a potentially unadjusted confounder that could explain away the observed associations [24]. The E-value is defined as the minimum strength that an unmeasured or uncontrolled confounder would have with both the exposure and the outcome to fully explain away the observed association. To take into account potentials undiagnosed disease, we conducted further sensitivity analyses by excluding the first 2 years of follow-up. The analyses were conducted using Stata13.0 statistical software. All *p*-values refer to two-tailed tests. Statistical significance was set at *p* < 0.05.

## Results

A total of 210,748 participants (92,483 men and 118,265 women) aged 18–84 years were included in the current analysis (Table 1). Participants with self-reported chronic kidney disease had lower education, lower income, higher BMI, lower levels of physical activity, and lower prevalence of current drinkers, when compared to participants without self-reported kidney disease (Table 1). There were little differences in prevalence of chronic kidney disease by sex or smoking status but more Hispanics and non-Hispanic blacks than whites or other ethnicities had prevalent chronic kidney disease at baseline (Table 1).

**Table 1:** Baseline characteristics of participants with and without chronic kidney disease

	No chronic kidney disease		Chronic kidney disease	
	N	Percentage	N	Percentage
Total	208,238	98.8	2510	1.2
Age (years)	43.5		50.4	
Sex				
Men	91,579	48.3	904	40.3
Women	116,659	51.7	1606	59.7
Race/ethnicity				
Hispanics	36,873	11.7	649	17.6
Non-Hispanic white	134,485	72.3	1331	63.4
Non-Hispanic black	29,325	11.7	459	15.7
Non-Hispanic other	7555	4.3	71	3.3
Education				
Less than high school	39,731	16.4	956	33.8
High school degree	59,981	29.7	679	29.3
More than high school	107,327	53.3	858	36.2
Missing	1199	0.6	17	0.7
Income				
Low	32,405	11.6	802	25.4
Middle	105,123	49	1374	57
High	70,710	39.4	334	17.6
BMI				
< 25	85,558	41.5	919	36.3
25- < 30	70,509	34	785	30.7
≥ 30	45,075	21.2	732	30.1
Missing	7096	3.3	74	3
Physical activity				
Inactive	79,404	35.7	1475	56.7
Insufficient activity	39,711	19.6	425	17.4
Sufficient activity	82,551	41.3	551	23.5
Missing	6572	3.4	59	2.4
Smoking status				
Never	115,931	55.6	1248	49.4
Former	41,198	20.1	590	24.3
Current	49,965	23.8	655	25.7
Missing	1144	0.5	17	0.5
Alcohol intake				
Never	47,364	21.9	759	28.9
Former	28,104	13	635	25.8
Current	129,877	63.7	1088	44.3
Missing	2893	1.4	28	1.1

During a total of 1,256,385 person-years of follow-up (mean follow-up: 6 years), 9564 deaths occurred. The most common causes of death were circulatory disease (*n* = 2976), cancer (*n* = 2183), ischemic heart disease (*n* = 1619), other chronic ischemic heart disease (*n* = 724), acute myocardial infarction (*n* = 635), lung cancer (*n* = 715), and cerebrovascular disease (*n* = 480) (Supplementary Table 2).

**Fig. 1:**
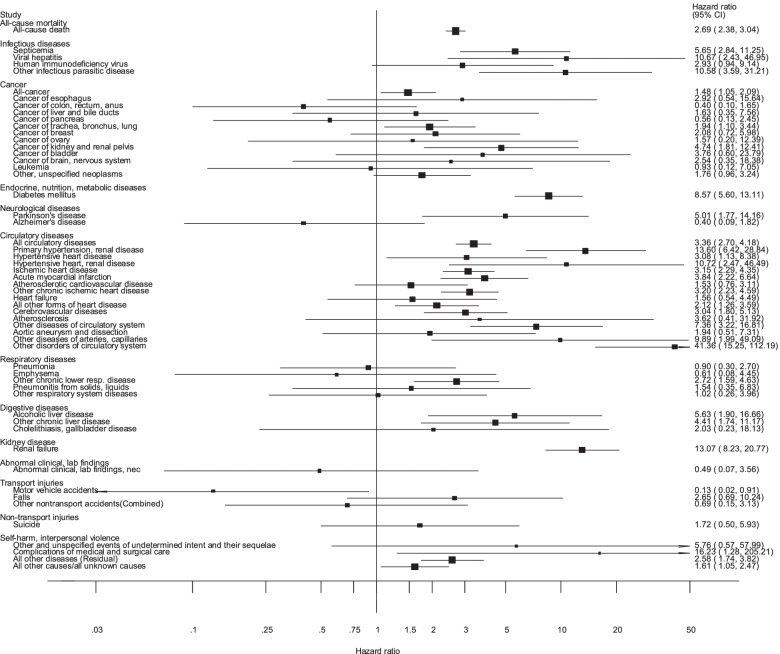
Hazard ratios and 95% confidence intervals for the association between kidney disease and all-cause and cause-specific mortality

Participants with chronic kidney disease vs. participants without the condition were at increased risk of all-cause mortality (HR = 2.69, 95% CI: 2.38–3.04), and mortality from septicemia (HR = 5.65, 95% CI: 2.84–11.25), viral hepatitis (HR = 10.67, 95% CI: 2.43–46.95), other infectious parasitic diseases (HR = 10.58, 95% CI: 3.59–31.21), total cancer (HR = 1.48, 95% CI: 1.05–2.09), lung cancer (HR = 1.94, 95% CI: 1.10–3.44), kidney cancer (HR = 4.74, 95% CI: 1.81–12.41) (Fig. 1, Supplementary Table 2). Participants with kidney disease were also at increased risk of mortality from diabetes mellitus (HR = 8.57, 95% CI: 5.60–13.11) and Parkinson’s disease (HR = 5.01, 95% CI: 1.77–14.16) (Supplementary Table 2). Elevated risks were also observed for mortality from a range of circulatory disease outcomes including all circulatory diseases combined (HR = 3.36, 95% CI: 2.70–4.18), primary hypertension/renal disease (HR = 13.60, 95% CI: 6.42–28.84), hypertensive heart disease (HR = 3.08, 95% CI: 1.13–8.38), hypertensive heart/renal disease (HR = 10.72, 95% CI: 2.47–46.49), ischemic heart disease (HR = 3.15, 95% CI: 2.29–4.35), acute myocardial infarction (HR = 3.84, 95% CI: 2.22–6.64), other chronic ischemic heart disease (HR = 3.20, 95% CI: 2.23–4.59), all other forms of heart disease (HR = 2.12, 95% CI: 1.26–3.59), cerebrovascular disease (HR = 3.04, 95% CI: 1.80–5.13), other diseases of circulatory system (HR = 7.36, 95% CI: 3.22–16.81), other diseases of arteries or capillaries (HR = 9.89, 95% CI: 1.99–49.09), and other disorders of circulatory system (HR = 41.36, 95% CI: 15.25–112.19) (Supplementary Table 2). In addition, increased risk was observed for several other outcomes including other chronic lower respiratory disease (HR = 2.72, 95% CI: 1.59–4.63), alcoholic liver disease (HR = 5.63, 95% CI: 1.90–16.66), other chronic liver disease (HR = 4.41, 95% CI: 1.74–11.17), kidney failure (HR = 13.07, 95% CI: 8.23–20.77), complications of medical/surgical care (HR = 16.23, 95% CI: 1.28–205.21), all other diseases (HR = 2.58, 95% CI: 1.74–3.82), and all other causes/all unknown causes (HR = 1.61, 95% CI: 1.05–2.47) (Supplementary Table 2).

In sensitivity analyses excluding first 2 years of follow-up these associations were not substantially altered (Supplementary Table 3). In addition, the elevated risk of all-cause mortality as well as cause-specific mortality persisted, in general, across strata of age, sex, race/ethnicity, BMI, physical activity and smoking (Supplementary Table 4-9).

The estimated E-values were 4.82 (lower CI: 4.19) for all-cause mortality, 10.77 (lower CI: 5.12) for septicemia, 20.83 (lower CI: 4.29) for viral hepatitis, 20.65 (lower CI: 6.63) for other infectious parasitic diseases, 2.32 (lower CI: 1.28) for all cancers, 3.30 (lower CI: 1.42) for lung cancer, 8.95 (lower CI: 3.02) for kidney cancer, 16.62 (lower CI: 10.67) for diabetes mellitus, 6.18 (lower CI: 4.84) for circulatory diseases, 26.70 (lower CI: 12.31) for primary hypertension/renal disease, 20.92 (lower CI: 4.38) for hypertensive heart/renal disease, 19.27 (lower CI: 3.40) for other diseases of arteries or capillaries, 82.22 (lower CI: 29.99) for other disorders of the circulatory system, 25.63 (lower CI: 15.94) for kidney failure, 31.96 (lower CI: 1.89) for complications of medical/surgical care (Supplementary Table 10).

## Discussion

The current outcome-wide mortality analysis found a 2.7-fold increase in all-cause mortality among participants with self-reported chronic kidney disease compared to those without chronic kidney disease and in addition, increased risk was observed for 27 out of 54 specific causes of death investigated. Thirteen of these causes of death were circulatory diseases, with particularly strong associations (10–41-fold increases) observed for mortality from primary hypertension and renal disease, hypertensive heart and renal disease, and other disorders of the circulatory system, and an 8-fold increase was observed for diabetes mortality and a 13-fold increase was observed in mortality from renal failure. Increased risk was also observed for mortality from septicemia, viral hepatitis, other infectious parasitic disease, total cancer, lung and kidney cancer, Parkinson’s disease, other chronic lower respiratory disease, alcoholic liver disease, other chronic liver disease, and complications of medical/surgical care, and all other diseases, and all other causes of death. Many of the results persisted across strata of age, sex, race/ethnicity, BMI, physical activity and smoking.

We found strong positive associations between chronic kidney disease and risk of mortality from septicemia, viral hepatitis, and other infectious parasitic disease and a non-significant association HIV mortality. The finding on sepsis is consistent with previous studies showing poorer survival in sepsis patients with chronic kidney disease [25, 26] and with studies showing an increased risk of infections and sepsis in chronic kidney disease patients [20, 21, 27, 28]. The positive association with total cancer mortality and strong positive association with kidney cancer mortality is consistent with a Taiwanese cohort study [21], and a pooled analysis of six cohorts which reported an increased risk of urinary tract cancers in chronic kidney disease [29]. However, the positive association with lung cancer is inconsistent with previous studies [21, 29]. For other cancers we had limited statistical power to detect any associations because of few deaths among the modest number of participants reporting a chronic kidney disease diagnosis. However, biological plausibility is likely strongest for kidney and urinary tract cancers. The strong positive association between chronic kidney disease and diabetes mortality is consistent with previous studies [20, 21], but we are not aware of any previous studies on mortality from Parkinson’s disease.

We found an increased risk of 12 out of 16 circulatory disease mortality outcomes among chronic kidney disease patients. Although previous studies have established an association between chronic kidney disease and increased cardiovascular disease risk [4, 5, 11, 15–17, 19], we are not aware of any previous studies that have investigated such a broad range of circulatory disease outcomes as the current study. The kidneys play an important role in blood pressure regulation through the renin-angiotensin-aldosterone axis, and impairment of kidney function can cause hypertension, which is an important risk factor for a range of cardiovascular outcomes [30, 31]. Although chronic kidney disease and circulatory diseases share common risk factors such as smoking, adiposity and physical activity, the observed associations persisted in general across strata of age, sex, race/ethnicity, BMI, physical activity, and smoking status, suggesting associations largely independent of these risk factors.

There was a positive association with chronic lower respiratory disease, but not with other respiratory disease outcomes, while previous studies have reported mixed results [20, 21]. A positive association was also observed for alcoholic liver disease mortality, and a previous study reported a HR of 6.84 (0.95–49.24) for cirrhosis mortality for the lowest versus highest category of estimated glomerular filtration rate, although no association was observed with chronic kidney disease as a dichotomous variable [21]. The strong positive association with kidney failure mortality is consistent with a previous study [20] and as expected. The increased risk of mortality from suicide is consistent with previous studies [32–34], and is likely explained by poor quality of life and depression related to severe chronic kidney disease and dialysis [35]. We also found strong associations with mortality due to complications of medical/surgical care, all other diseases, and all other causes/unknown causes of death, but some of these associations were based on few deaths in the chronic kidney disease patients.

One limitation of our study is that we relied on self-reported data at baseline on chronic kidney disease rather than objective measures of kidney function, such as estimated glomerular filtration rate or proteinurea. We were therefore not able to assess the impact of different levels of kidney function as in some previous studies. Use of self-reported data is likely to have led to underestimation of the number of participants with chronic kidney disease, but would most likely lead to underestimation of the observed associations. The baseline questionnaire asked about chronic kidney disease diagnosed in the past 12 months prior to baseline, and cases that occurred > 12 months before baseline may therefore not have been captured and this could have biased the results toward the null, under the assumption that longer duration of chronic kidney disease may be more strongly associated with mortality than shorter duration. We were also not able to assess the association between end-stage renal disease or kidney transplantation and mortality because of lack of a data. Because we did not have data on incident chronic kidney disease, we were not able to take into account changes in chronic kidney disease status during follow-up, however, given the prospective design it is possible that such misclassification would have been non-differential and may have biased the associations toward the null. Although we adjusted for several important confounding factors (age, sex, education, income, alcohol, smoking, BMI, physical activity and survey year) we cannot completely rule out the potential for residual confounding from unmeasured confounders. However, the observed associations persisted across categories of smoking, BMI and physical activity which might suggest that the observed associations are likely independent of these risk factors. The estimated E-values also suggest that the unobserved confounder(s) would have to be strongly associated with chronic kidney disease and mortality to completely explain away the observed associations. The duration of follow-up was relatively short and the number of deaths was modest so we may not have had sufficient power to detect significant associations across all causes of death, however, the findings are still important as an indicator of outcomes that may need further study in other cohorts. Lastly, we did not have information available on all prevalent diseases at baseline, thus we were not able to exclude participants with prevalent disease across all causes of death examined.

Strengths of this study include the prospective design and relatively large nationally representative sample which allowed for analyses of much more detailed causes of death than most previous studies, including many causes of death that have been minimally studied previously. We adjusted for important confounding factors and the results persisted in a number of stratified analyses, as well as in analyses excluding early follow-up. Many of the observed results, particularly for cardiovascular outcomes, are consistent with previous studies, lending some credibility to the observed associations.

This analysis found that a self-reported chronic kidney disease was associated with increased risk of all-cause mortality as well as with mortality from 27 out of 54 specific causes of death that were examined. Many of these mortality outcomes were circulatory, metabolic, infectious and renal disease outcomes. Additional studies are needed to explore these associations with more precision, but the wide range of adverse associations suggest increased emphasis should be placed on identifying further risk factors for kidney disease and on primary prevention.

## Supplementary Information

**Additional file 1: Supplementary Table 1.** List of ICD codes for cause-specific mortality. **Supplementary Table 2.** Hazard ratios and 95% confidence intervals (CIs) of all-cause mortality and cause-specific mortality among participants with chronic kidney disease vs. no chronic kidney disease. **Supplementary Table 3.** Hazard ratios and 95% confidence intervals (CIs) of all-cause mortality and cause-specific mortality among participants with chronic kidney disease vs. no chronic kidney disease, sensitivity analysis  excluding first 2 years of follow-up. **Supplementary Table 4.** Hazard ratios and 95% confidence intervals (CIs) of all-cause mortality and cause-specific mortality among participants with chronic kidney disease vs. no chronic kidney disease, stratified by age. **Supplementary Table 5.** Hazard ratios and 95% confidence intervals (CIs) of all-cause mortality and cause-specific mortality among participants with chronic kidney disease vs. no chronic kidney disease, stratified by sex. **Supplementary Table 6.** Hazard ratios and 95% confidence intervals (CIs) of all-cause mortality and cause-specific mortality among participants with chronic kidney disease vs. no chronic kidney disease, stratified by race/ethnicity. **Supplementary Table 7.** Hazard ratios and 95% confidence intervals (CIs) of all-cause mortality and cause-specific mortality among participants with chronic kidney disease vs. no chronic kidney disease, stratified by BMI. **Supplementary Table 8.** Hazard ratios and 95% confidence intervals (CIs) of all-cause mortality and cause-specific mortality among participants with chronic kidney disease vs. no chronic kidney disease, stratified by physical activity. . **Supplementary Table 9.** Hazard ratios and 95% confidence intervals (CIs) of all-cause mortality and cause-specific mortality among participants with chronic kidney disease vs. no chronic kidney disease, stratified by smoking status. **Supplementary Table 10.** Hazard ratios and 95% confidence intervals (CIs) of all-cause mortality and cause-specific mortality among participants with chronic kidney disease vs. no chronic kidney disease and corresponding E-values.

## Data Availability

Data from the study are freely available online at the following website https://www.cdc.gov/nchs/nhis/index.htm
