# Supplementary material for: Self-reported chronic kidney disease and the risk of all-cause and cause-specific mortality: outcome-wide association study of 54 causes of death in the National Health Interview Survey
Source: BMC Nephrol. 2022 Apr 30;23:165. doi: 10.1186/s12882-022-02771-1 (PMC9055730; doi:10.1186/s12882-022-02771-1)
Supplement: Supplementary file 1 — Additional file 1: Supplementary Table 1. List of ICD codes for cause-specific mortality. Supplementary Table 2. Hazard ratios and 95% confidence intervals (CIs) of all-cause mortality and cause-specific mortality among participants with chronic kidney disease vs. no chronic kidney disease. Supplementary Table 3. Hazard ratios and 95% confidence intervals (CIs) of all-cause mortality and cause-specific mortality among participants with chronic kidney disease vs. no chronic kidney disease, sensitivity analysis excluding first 2 years of follow-up. Supplementary Table 4. Hazard ratios and 95% confidence intervals (CIs) of all-cause mortality and cause-specific mortality among participants with chronic kidney disease vs. no chronic kidney disease, stratified by age. Supplementary Table 5. Hazard ratios and 95% confidence intervals (CIs) of all-cause mortality and cause-specific mortality among participants with chronic kidney disease vs. no chronic kidney disease, stratified by sex. Supplementary Table 6. Hazard ratios and 95% confidence intervals (CIs) of all-cause mortality and cause-specific mortality among participants with chronic kidney disease vs. no chronic kidney disease, stratified by race/ethnicity. Supplementary Table 7. Hazard ratios and 95% confidence intervals (CIs) of all-cause mortality and cause-specific mortality among participants with chronic kidney disease vs. no chronic kidney disease, stratified by BMI. Supplementary Table 8. Hazard ratios and 95% confidence intervals (CIs) of all-cause mortality and cause-specific mortality among participants with chronic kidney disease vs. no chronic kidney disease, stratified by physical activity. . Supplementary Table 9. Hazard ratios and 95% confidence intervals (CIs) of all-cause mortality and cause-specific mortality among participants with chronic kidney disease vs. no chronic kidney disease, stratified by smoking status. Supplementary Table 10. Hazard ratios and 95% confidence intervals (CIs) of all-cau [file 12882_2022_2771_MOESM1_ESM.docx]

Supplementary Table 1. List of ICD codes for cause-specific mortality

| Cause of death (ICD-10 code) |  |
| --- | --- |
| Infections |  |
| Septicemia | A40-A41 |
| Viral hepatitis | B15-B19 |
| Human immunodeficiency virus | B20-B24 |
| Other infectious and parasitic disease | A00,A05,A20-A36,A42- A44,A48-A49,A54-A79,A81-A82,A85.0-A85.1,A85.8, A86-B04,B06-B09,B25-B49,B55-B99 |
| Cancers |  |
| All cancers | C00-C95, C97 |
| Esophagus | C15 |
| Colon, rectum, anus | C18-C21 |
| Liver and bile ducts | C22 |
| Pancreas | C25 |
| Lung, trachea, bronchus | C33-C34 |
| Breast females | C50 |
| Ovaries females | C56 |
| Kidney and renal pelvis | C64-C65 |
| Bladder | C67 |
| Brain, nervous system | C70-C72 |
| Leukemia | C91-C95 |
| All other and unspecified neoplasms | C00-C14, C16, C17,C23-C24,C26-C31, C32, C37-C41, C43, C44-C49,C51-C52, C53, C54, C55, C57-C60, C61, C62-C63,C66,C68-C69,C73-C80, C81, C82-C85, C88-C90, C97 |
| Endocrine, nutritional, metabolic diseases |  |
| Diabetes mellitus | E10-E14 |
| Nervous system |  |
| Parkinson's disease | G20-G21 |
| Alzheimer's disease | G30 |
| Circulatory disease |  |
| All circulatory diseases | I10-I13, I20-I25, I26-I28, I34-I38, I42-I49, I50, I51, I60-I69, I70-I78, I80-I99 |
| Primary hypertension and hypertensive renal disease | I10, I12 |
| Hypertensive heart disease | I11 |
| Hypertensive heart and renal disease | I13 |
| Ischaemic heart disease | I20-I25 |
| Acute myocardial infarction | I21-I22 |
| Atherosclerotic cardiovascular disease | I25.0 |
| Other chronic ischaemic heart disease | I20, I25.1-I25.9 |
| Heart failure | I50 |
| All other forms of heart disease | I26-I28,I34-I38,I42-I49,I51 |
| Cerebrovascular disease | I60-I69 |
| Atherosclerosis | I70 |
| Other diseases of circulatory system | I71-I78 |
| Aortic aneurysm and dissection | I71 |
| Other diseases of arteries or capillaries | I72-I78 |
| Other disorders of circulatory system | I80-I99 |
| Respiratory diseases |  |
| Pneumonia | J12-J18 |
| Emphysema | J43 |
| Other chronic lower respiratory disease | J44, J47 |
| Pneumonitis from solids, liquids | J69 |
| Other respiratory system diseases | J00-J06, J30-J39, J67, J70-J98 |
| Digestive diseases |  |
| Alcoholic liver disease | K70 |
| Other chronic liver disease | K73-K74 |
| Cholelithiasis, gallbladder disease | K80-K82 |
| Urinary tract disease |  |
| Kidney failure | N17-N19 |
| Abnormal clinical, lab findings | R00-R99 |
| Transport injuries |  |
| Motor vehicle accidents | V02-V04,V09.0,V09.2,V12-V14,V19.0-V19.2, V19.4-V19.6,V20-V79,V80.3-V80.5,V81.0-V81.1,V82.0-V82.1,V83-V86, V87.0-V87.8,V88.0-V88.8,V89.0,V89.2 |
| Unintentional injuries |  |
| Falls | W00-W19 |
| Other non-transport accidents combined | W20-X59,Y86 |
| Self-harm, interpersonal violence |  |
| Suicide | X60-X84, Y87.0 |
| Other causes |  |
| Other and unspecified events of  undetermined intent and their sequelae | Y10-Y21,Y25-Y34,Y87.2,Y89.9 |
| Complications of medical/surgical care | Y40-Y84,Y88 |
| All other diseases (residual ) | D50-D64, D65-E07,E15-E34, E40-E46, E65-F99,G04-G12,G23-G25, G31-H93,K00- K22,K29-K31,K50-K66,K71-K72,K75-K76,K83-M99,N13.0-N13.5, N13.7-N13.9,N14,N15.0,N15.8- N15.9,N20-N23,N28-N39,N41-N64,N80-N98 |
| All other causes/all unknown causes | A01-A02, A03,A06, A04, A07-A09, A16-A19, A37, A38, A46, A39, A50-A53, A80, A83-A84, A85.2, B05, B50-B54, C96, E50-E64, G00, G03, I00-I09, I33, I30-I31, I40, J10-J11, J20-J21, J22, J40-J42, J45-J46, J60-J66, J68, K25-K28, K35-K38, K40-K46, N00-N07, N10-N12, N13.6, N15.1, N25-N27, N40, N70-N76, O00-O99, P00-P96, Q00-Q99, V01, V05-V06, V09.1, V09.3-V09.9, V10-V11, V15-V18, V19.3, V19.8-V19.9, V80.0-V80.2, V80.6-V80.9, V81.2-V81.9, V82.2-V82.9, V87.9, V88.9, V89.1, V89.3, V89.9, V90-V99, W32-W34, X85-Y09, Y22-Y24, Y85, Y87.1, Y89.0, Y89.1 and unknown causes. |

Supplementary Table 2. Hazard ratios and 95% confidence intervals (CIs) of all-cause mortality and cause-specific mortality among participants with chronic kidney disease vs. no chronic kidney disease

|  | Total | No chronic kidney disease | | Chronic kidney disease | |  |
| --- | --- | --- | --- | --- | --- | --- |
|  | N  (deaths) | N  (deaths) | HR | N (deaths) | HR (95% CI) | P-value |
| All-cause mortality | 9564 | 9122 | 1.00 | 442 | 2.69 (2.38-3.04) | <0.001 |
| Infections |  |  |  |  |  |  |
| Septicemia | 130 | 117 | 1.00 | 13 | 5.65 (2.84-11.25) | <0.001 |
| Viral hepatitis | 28 | 26 | 1.00 | 2 | 10.67 (2.43-46.95) | <0.001 |
| Human immunodeficiency virus | 72 | 68 | 1.00 | 4 | 2.93 (0.94-9.14) | 0.06 |
| Other infectious parasitic disease | 30 | 25 | 1.00 | 5 | 10.58 (3.59-31.21) | <0.001 |
| Cancers |  |  |  |  |  |  |
| All cancers | 2183 | 2136 | 1.00 | 47 | 1.48 (1.05-2.09) | 0.03 |
| Esophagus | 54 | 52 | 1.00 | 2 | 2.92 (0.54-15.64) | 0.21 |
| Colon, rectum, anus | 188 | 186 | 1.00 | 2 | 0.40 (0.10-1.65) | 0.20 |
| Liver and bile ducts | 65 | 63 | 1.00 | 2 | 1.63 (0.35-7.56) | 0.54 |
| Pancreas | 136 | 134 | 1.00 | 2 | 0.56 (0.13-2.45) | 0.44 |
| Lung, trachea, bronchus | 715 | 697 | 1.00 | 18 | 1.94 (1.10-3.44) | 0.02 |
| Breast (females) | 119 | 115 | 1.00 | 4 | 2.08 (0.72-5.98) | 0.17 |
| Ovaries (females) | 58 | 57 | 1.00 | 1 | 1.57 (0.20-12.39) | 0.67 |
| Kidney and renal pelvis | 51 | 46 | 1.00 | 5 | 4.74 (1.81-12.41) | <0.001 |
| Bladder | 42 | 41 | 1.00 | 1 | 3.76 (0.60-23.79) | 0.16 |
| Brain, nervous system | 39 | 38 | 1.00 | 1 | 2.54 (0.35-18.38) | 0.36 |
| Leukemia | 80 | 79 | 1.00 | 1 | 0.93 (0.12-7.05) | 0.95 |
| Other, unspecified neoplasms | 435 | 423 | 1.00 | 12 | 1.76 (0.96-3.24) | 0.07 |
| Endocrine, nutritional, metabolic diseases |  |  |  |  |  |  |
| Diabetes mellitus | 288 | 247 | 1.00 | 41 | 8.57 (5.60-13.11) | <0.001 |
| Nervous system |  |  |  |  |  |  |
| Parkinson's disease | 53 | 49 | 1.00 | 4 | 5.01 (1.77-14.16) | <0.001 |
| Alzheimer's disease | 140 | 138 | 1.00 | 2 | 0.40 (0.09-1.82) | 0.24 |
| Circulatory disease |  |  |  |  |  |  |
| All circulatory diseases | 2976 | 2804 | 1.00 | 172 | 3.36 (2.70-4.18) | <0.001 |
| Primary hypertension, renal disease | 84 | 70 | 1.00 | 14 | 13.60 (6.42-28.84) | <0.001 |
| Hypertensive heart disease | 109 | 103 | 1.00 | 6 | 3.08 (1.13-8.38) | 0.03 |
| Hypertensive heart, renal disease | 14 | 11 | 1.00 | 3 | 10.72 (2.47-46.49) | <0.001 |
| Ischaemic heart disease | 1619 | 1524 | 1.00 | 95 | 3.15 (2.29-4.35) | <0.001 |
| Acute myocardial infarction | 635 | 595 | 1.00 | 40 | 3.84 (2.22-6.64) | <0.001 |
| Atherosclerotic cardiovascular disease | 246 | 237 | 1.00 | 9 | 1.53 (0.76-3.11) | 0.24 |
| Other chronic ischaemic heart disease | 724 | 678 | 1.00 | 46 | 3.20 (2.23-4.59) | <0.001 |
| Heart failure | 147 | 143 | 1.00 | 4 | 1.56 (0.54-4.49) | 0.41 |
| All other forms of heart disease | 381 | 365 | 1.00 | 16 | 2.12 (1.26-3.59) | 0.01 |
| Cerebrovascular disease | 480 | 457 | 1.00 | 23 | 3.04 (1.80-5.13) | <0.001 |
| Atherosclerosis | 17 | 16 | 1.00 | 1 | 3.62 (0.41-31.92) | 0.25 |
| Other diseases of circulatory system | 125 | 115 | 1.00 | 10 | 7.36 (3.22-16.81) | <0.001 |
| Aortic aneurysm and dissection | 65 | 62 | 1.00 | 3 | 1.94 (0.51-7.31) | 0.33 |
| Other diseases of arteries or capillaries | 42 | 39 | 1.00 | 3 | 9.89 (1.99-49.09) | 0.01 |
| Other disorders of circulatory system | 18 | 14 | 1.00 | 4 | 41.36 (15.25-112.19) | <0.001 |
| Respiratory diseases |  |  |  |  |  |  |
| Pneumonia | 177 | 173 | 1.00 | 4 | 0.90 (0.30-2.70) | 0.85 |
| Emphysema | 57 | 56 | 1.00 | 1 | 0.61 (0.08-4.45) | 0.63 |
| Other chronic lower respiratory disease | 425 | 403 | 1.00 | 22 | 2.72 (1.59-4.63) | <0.001 |
| Pneumonitis from solids, liquids | 60 | 58 | 1.00 | 2 | 1.54 (0.35-6.83) | 0.57 |
| Other respiratory system diseases | 115 | 112 | 1.00 | 3 | 1.02 (0.26-3.96) | 0.98 |
| Digestive diseases |  |  |  |  |  |  |
| Alcoholic liver disease | 80 | 75 | 1.00 | 5 | 5.63 (1.90-16.66) | <0.001 |
| Other chronic liver disease | 85 | 79 | 1.00 | 6 | 4.41 (1.74-11.17) | <0.001 |
| Cholelithiasis, gallbladder disease | 10 | 9 | 1.00 | 1 | 2.03 (0.23-18.13) | 0.53 |
| Urinary tract disease |  |  |  |  |  |  |
| Kidney failure | 154 | 121 | 1.00 | 33 | 13.07 (8.23-20.77) | <0.001 |
| Abnormal clinical, lab findings | 89 | 88 | 1.00 | 1 | 0.49 (0.07-3.56) | 0.48 |
| Transport injuries |  |  |  |  |  |  |
| Motor vehicle accidents | 167 | 166 | 1.00 | 1 | 0.13 (0.02-0.91) | 0.04 |
| Unintentional injuries |  |  |  |  |  |  |
| Falls | 63 | 60 | 1.00 | 3 | 2.65 (0.69-10.24) | 0.16 |
| Other non-transport accidents combined | 153 | 151 | 1.00 | 2 | 0.69 (0.15-3.13) | 0.63 |
| Self-harm, interpersonal violence |  |  |  |  |  |  |
| Suicide | 147 | 144 | 1.00 | 3 | 1.72 (0.50-5.93) | 0.39 |
| Other causes |  |  |  |  |  |  |
| Other and unspecified events of  undetermined intent and their sequelae | 11 | 10 | 1.00 | 1 | 5.76 (0.57-57.99) | 0.14 |
| Complications of medical/surgical care | 8 | 7 | 1.00 | 1 | 16.23 (1.28-205.21) | 0.03 |
| All other diseases (residual) | 757 | 723 | 1.00 | 34 | 2.58 (1.74-3.82) | <0.001 |
| All other causes/all unknown causes | 1095 | 1066 | 1.00 | 29 | 1.61 (1.05-2.47) | 0.03 |

Multivariable adjustment for age, sex, education, race, income, alcohol, smoking status, BMI, physical activity, and survey year

Supplementary Table 3. Hazard ratios and 95% confidence intervals (CIs) of all-cause mortality and cause-specific mortality among participants with chronic kidney disease vs. no chronic kidney disease, excluding first 2 years of follow-up

|  | Total | No chronic kidney disease | | Chronic kidney disease | |  |
| --- | --- | --- | --- | --- | --- | --- |
|  | N  (deaths) | N  (deaths) | HR | N (deaths) | HR (95% CI) | P-value |
| All-cause mortality | 7416 | 7118 | 1.00 | 298 | 2.43 (2.10-2.82) | <0.001 |
| Infections |  |  |  |  |  |  |
| Septicemia | 104 | 95 | 1.00 | 9 | 4.80 (1.97-11.72) | <0.001 |
| Viral hepatitis | 19 | 18 | 1.00 | 1 | 6.14 (0.71-53.49) | 0.10 |
| Human immunodeficiency virus | 55 | 53 | 1.00 | 2 | 2.41 (0.51-11.31) | 0.27 |
| Other infectious parasitic disease | 21 | 18 | 1.00 | 3 | 11.17 (2.74-45.45) | <0.001 |
| Cancers |  |  |  |  |  |  |
| All cancers | 1772 | 1735 | 1.00 | 37 | 1.40 (0.96-2.04) | 0.08 |
| Esophagus | 46 | 44 | 1.00 | 2 | 3.90 (0.73-20.87) | 0.11 |
| Colon, rectum, anus | 154 | 152 | 1.00 | 2 | 0.52 (0.12-2.14) | 0.36 |
| Liver and bile ducts | 52 | 51 | 1.00 | 1 | 1.42 (0.19-10.48) | 0.73 |
| Pancreas | 105 | 104 | 1.00 | 1 | 0.46 (0.06-3.50) | 0.45 |
| Lung, trachea, bronchus | 577 | 565 | 1.00 | 12 | 1.41 (0.72-2.74) | 0.32 |
| Breast (females) | 98 | 95 | 1.00 | 3 | 1.64 (0.51-5.26) | 0.40 |
| Ovaries (females) | 44 | 43 | 1.00 | 1 | 2.09 (0.26-16.96) | 0.49 |
| Kidney and renal pelvis | 45 | 40 | 1.00 | 5 | 5.71 (2.17-15.02) | <0.001 |
| Bladder | 37 | 36 | 1.00 | 1 | 4.23 (0.75-23.80) | 0.10 |
| Brain, nervous system | 31 | 30 | 1.00 | 1 | 3.41 (0.47-24.80) | 0.22 |
| Leukemia | 58 | 58 | 1.00 | 0 | - | - |
| Other, unspecified neoplasms | 354 | 343 | 1.00 | 11 | 2.09 (1.10-3.94) | 0.02 |
| Endocrine, nutritional, metabolic diseases |  |  |  |  |  |  |
| Diabetes mellitus | 226 | 202 | 1.00 | 24 | 5.41 (3.16-9.28) | <0.001 |
| Nervous system |  |  |  |  |  |  |
| Parkinson's disease | 44 | 40 | 1.00 | 4 | 6.85 (2.28-20.63) | <0.001 |
| Alzheimer's disease | 125 | 123 | 1.00 | 2 | 0.53 (0.12-2.39) | 0.41 |
| Circulatory disease |  |  |  |  |  |  |
| All circulatory diseases | 2279 | 2167 | 1.00 | 112 | 3.22 (2.44-4.25) | <0.001 |
| Primary hypertension, renal disease | 72 | 61 | 1.00 | 11 | 14.40(6.28-33.00) | <0.001 |
| Hypertensive heart disease | 82 | 79 | 1.00 | 3 | 2.26 (0.47-10.96) | 0.31 |
| Hypertensive heart, renal disease | 9 | 9 | 1.00 | 0 | - | - |
| Ischaemic heart disease | 1229 | 1165 | 1.00 | 64 | 3.16 (2.09-4.77) | <0.001 |
| Acute myocardial infarction | 460 | 434 | 1.00 | 26 | 4.09 (2.00-8.34) | <0.001 |
| Atherosclerotic cardiovascular disease | 184 | 178 | 1.00 | 6 | 1.59 (0.67-3.74) | 0.29 |
| Other chronic ischaemic heart disease | 574 | 542 | 1.00 | 32 | 3.02 (1.97-4.61) | <0.001 |
| Heart failure | 123 | 119 | 1.00 | 4 | 2.00 (0.69-5.81) | 0.20 |
| All other forms of heart disease | 287 | 273 | 1.00 | 14 | 2.86 (1.60-5.09) | <0.001 |
| Cerebrovascular disease | 380 | 368 | 1.00 | 12 | 1.89 (0.98-3.64) | 0.06 |
| Atherosclerosis | 14 | 13 | 1.00 | 1 | 5.11 (0.59-44.12) | 0.14 |
| Other diseases of circulatory system | 83 | 80 | 1.00 | 3 | 6.05 (1.63-22.49) | 0.01 |
| Aortic aneurysm and dissection | 39 | 39 | 1.00 | 0 | - | - |
| Other diseases of arteries or capillaries | 33 | 32 | 1.00 | 1 | 7.52 (0.81-69.66) | 0.08 |
| Other disorders of circulatory system | 11 | 9 | 1.00 | 2 | - | - |
| Respiratory diseases |  |  |  |  |  |  |
| Pneumonia | 146 | 142 | 1.00 | 4 | 1.19 (0.39-3.58) | 0.76 |
| Emphysema | 43 | 43 | 1.00 | 0 | - | - |
| Other chronic lower respiratory disease | 333 | 317 | 1.00 | 16 | 2.71 (1.48-4.95) | <0.001 |
| Pneumonitis from solids, liquids | 51 | 49 | 1.00 | 2 | 2.11 (0.47-9.52) | 0.33 |
| Other respiratory system diseases | 92 | 91 | 1.00 | 1 | 0.15 (0.02-1.10) | 0.06 |
| Digestive diseases |  |  |  |  |  |  |
| Alcoholic liver disease | 57 | 54 | 1.00 | 6 | 4.58 (1.33-15.82) | 0.02 |
| Other chronic liver disease | 67 | 64 | 1.00 | 3 | 2.4 (0.69-8.43) | 0.17 |
| Cholelithiasis, gallbladder disease | 10 | 9 | 1.00 | 1 | 2.03 (0.23-18.13) | 0.53 |
| Urinary tract disease |  |  |  |  |  |  |
| Kidney failure | 124 | 99 | 1.00 | 25 | 12.59 (7.30-21.69) | <0.001 |
| Abnormal clinical, lab findings | 66 | 66 | 1.00 | 0 | - | - |
| Transport injuries |  |  |  |  |  |  |
| Motor vehicle accidents | 116 | 115 | 1.00 | 1 | 0.17 (0.02-1.28) | 0.09 |
| Unintentional injuries |  |  |  |  |  |  |
| Falls | 48 | 45 | 1.00 | 3 | 3.67 (0.93-14.53) | 0.06 |
| Other non-transport accidents combined | 113 | 111 | 1.00 | 2 | 1.03 (0.23-4.66) | 0.97 |
| Self-harm, interpersonal violence |  |  |  |  |  |  |
| Suicide | 90 | 87 | 1.00 | 3 | 3.31 (0.98-11.16) | 0.05 |
| Other causes |  |  |  |  |  |  |
| Other and unspecified events of  undetermined intent and their sequelae | 8 | 7 | 1.00 | 1 | 12.90 (1.46-114.10) | 0.02 |
| Complications of medical/surgical care | 5 | 4 | 1.00 | 1 | 37.59 (2.27-621.68) | 0.01 |
| All other diseases (residual) | 598 | 579 | 1.00 | 19 | 1.96 (1.13-3.40) | 0.02 |
| All other causes/all unknown causes | 794 | 778 | 1.00 | 19 | 1.42 (0.84-2.40) | 0.20 |

Multivariable adjustment for age, sex, education, race, income, alcohol, smoking status, BMI, physical activity, and survey year

Supplementary Table 4. Hazard ratios and 95% confidence intervals (CIs) of all-cause mortality and cause-specific mortality among participants with chronic kidney disease vs. no chronic kidney disease, stratified by age

|  |  | Total | No chronic kidney disease | | Chronic kidney disease | |  |  |
| --- | --- | --- | --- | --- | --- | --- | --- | --- |
|  | Age | N  (deaths) | N  (deaths) | HR | N (deaths) | HR (95% CI) | P-value | P-value for interaction |
| All-cause mortality | <55 | 2661 | 2540 | 1.00 | 121 | 4.47 (3.56-5.61) | <0.001 | <0.001 |
|  | ≥55 | 6903 | 6582 | 1.00 | 321 | 2.34 (2.02-2.71) | <0.001 |  |
| Infections |  |  |  |  |  |  |  |  |
| Septicemia | <55 | 27 | 23 | 1.00 | 4 | 18.59 (5.33-64.85) | <0.001 | 0.04 |
|  | ≥55 | 103 | 94 | 1.00 | 9 | 3.52 (1.58-7.84) | <0.001 |  |
| Viral hepatitis | <55 | 17 | 15 | 1.00 | 2 | 23.63 (4.67-119.44) | <0.001 | - |
|  | ≥55 | 11 | 11 | 1.00 | 0 | - | - |  |
| Human immunodeficiency virus | <55 | 60 | 56 | 1.00 | 4 | 4.76 (1.53-14.81) | 0.01 | - |
|  | ≥55 | 12 | 12 | 1.00 | 0 | - | - |  |
| Other infectious parasitic disease | <55 | 8 | 8 | 1.00 | 0 | - | - | - |
|  | ≥55 | 22 | 17 | 1.00 | 5 | 15.28 (5.39-43.31) | <0.001 |  |
| Cancers |  |  |  |  |  |  |  |  |
| All cancers | <55 | 488 | 476 | 1.00 | 12 | 2.50 (1.32-4.74) | 0.01 | 0.09 |
|  | ≥55 | 1695 | 1660 | 1.00 | 35 | 1.26 (0.85-1.87) | 0.26 |  |
| Lung, trachea, bronchus | <55 | 128 | 127 | 1.00 | 1 | 1.28 (0.17-9.39) | 0.81 | 0.67 |
|  | ≥55 | 587 | 570 | 1.00 | 17 | 1.92 (1.06-3.48) | 0.03 |  |
| Kidney and renal pelvis | <55 | 21 | 18 | 1.00 | 3 | 10.64 (3.05-37.17) | <0.001 | 0.17 |
|  | ≥55 | 30 | 28 | 1.00 | 2 | 1.98 (0.45-8.72) | 0.36 |  |
| Other, unspecified neoplasms | <55 | 127 | 121 | 1.00 | 6 | 4.47 (1.79-11.16) | 0.001 | 0.02 |
|  | ≥55 | 308 | 302 | 1.00 | 6 | 1.08 (0.46-2.53) | 0.86 |  |
| Endocrine, nutritional, metabolic diseases |  |  |  |  |  |  |  |  |
| Diabetes mellitus | <55 | 56 | 42 | 1.00 | 14 | 35.92（17.51-73.70） | <0.001 | <0.001 |
|  | ≥55 | 232 | 205 | 1.00 | 27 | 5.42（3.34-8.78） | <0.001 |  |
| Nervous system |  |  |  |  |  |  |  |  |
| Parkinson's disease | <55 | 0 | 0 | 1.00 | 0 | - | - | - |
|  | ≥55 | 53 | 49 | 1.00 | 4 | 4.82 (1.53-15.17) | 0.01 |  |
| Alzheimer's disease | <55 | 1 | 1 | 1.00 | 0 | - | - | - |
|  | ≥55 | 139 | 137 | 1.00 | 2 | 0.40 (0.09-1.79) | 0.23 |  |
| Circulatory disease |  |  |  |  |  |  |  |  |
| All circulatory diseases | <55 | 518 | 483 | 1.00 | 35 | 7.29 (4.71-11.30) | <0.001 | <0.001 |
|  | ≥55 | 2458 | 2321 | 1.00 | 137 | 2.81 (2.19-3.61) | <0.001 |  |
| Primary hypertension, renal disease | <55 | 11 | 5 | 1.00 | 6 | 158.79 (31.49-800.79) | <0.001 | <0.001 |
|  | ≥55 | 73 | 65 | 1.00 | 8 | 5.79 (2.51-13.34) | <0.001 |  |
| Hypertensive heart disease | <55 | 35 | 33 | 1.00 | 2 | 4.32 (0.73-25.73) | 0.11 | 0.67 |
|  | ≥55 | 74 | 70 | 1.00 | 4 | 2.72 (0.82-9.04) | 0.10 |  |
| Hypertensive heart, renal disease | <55 | 2 | 1 | 1.00 | 1 | - | - | - |
|  | ≥55 | 12 | 10 | 1.00 | 2 | 5.79 (1.13-29.67) | 0.04 |  |
| Ischaemic heart disease | <55 | 269 | 252 | 1.00 | 17 | 5.79 (3.24-10.36) | <0.001 | 0.03 |
|  | ≥55 | 1350 | 1272 | 1.00 | 78 | 2.74 (1.89-4.00) | <0.001 |  |
| Acute myocardial infarction | <55 | 108 | 100 | 1.00 | 8 | 6.52 (2.94-14.49) | <0.001 | 0.20 |
|  | ≥55 | 527 | 495 | 1.00 | 32 | 3.39 (1.78-6.48) | <0.001 |  |
| Atherosclerotic cardiovascular disease | <55 | 56 | 55 | 1.00 | 1 | 2.44 (0.34-17.31) | 0.37 | 0.79 |
|  | ≥55 | 190 | 182 | 1.00 | 8 | 1.34 (0.64-2.83) | 0.44 |  |
| Other chronic ischaemic heart disease | <55 | 101 | 93 | 1.00 | 8 | 6.71 (2.64-17.04) | <0.001 | 0.03 |
|  | ≥55 | 623 | 585 | 1.00 | 38 | 2.69 (1.83-3.95) | <0.001 |  |
| Heart failure | <55 | 10 | 10 | 1.00 | 0 | - | - | - |
|  | ≥55 | 137 | 133 | 1.00 | 4 | 1.55 (0.54-4.43) | 0.41 |  |
| All other forms of heart disease | <55 | 98 | 95 | 1.00 | 3 | 3.09 (0.89-10.74) | 0.08 | 0.45 |
|  | ≥55 | 283 | 270 | 1.00 | 13 | 1.98 (1.11-3.53) | 0.02 |  |
| Cerebrovascular disease | <55 | 72 | 69 | 1.00 | 3 | 3.64 (1.06-12.41) | 0.04 | 0.62 |
|  | ≥55 | 408 | 388 | 1.00 | 20 | 2.95 (1.68-5.17) | <0.001 |  |
| Other diseases of circulatory system | <55 | 21 | 18 | 1.00 | 3 | 32.01 (5.53-185.40) | <0.001 | 0.05 |
|  | ≥55 | 104 | 97 | 1.00 | 7 | 4.80 (1.96-11.73) | <0.001 |  |
| Aortic aneurysm and dissection | <55 | 9 | 8 | 1.00 | 1 | 3.97 (0.42-37.87) | 0.23 | 0.46 |
|  | ≥55 | 56 | 54 | 1.00 | 2 | 1.77 (0.38-8.22) | 0.47 |  |
| Other diseases of arteries or capillaries | <55 | 7 | 6 | 1.00 | 1 | - | - | - |
|  | ≥55 | 35 | 33 | 1.00 | 2 | 3.58 (0.57-22.49) | 0.17 |  |
| Other disorders of circulatory system | <55 | 5 | 4 | 1.00 | 1 | - | - | - |
|  | ≥55 | 13 | 10 | 1.00 | 3 | - | - |  |
| Respiratory diseases |  |  |  |  |  |  |  |  |
| Pneumonia | <55 | 24 | 22 | 1.00 | 2 | - |  | - |
|  | ≥55 | 153 | 151 | 1.00 | 2 | - |  |  |
| Other chronic lower respiratory disease | <55 | 29 | 25 | 1.00 | 4 | 6.93 (1.69-28.35) | 0.01 | 0.02 |
|  | ≥55 | 396 | 378 | 1.00 | 18 | 2.28 (1.27-4.08) | 0.01 |  |
| Pneumonitis from solids, liquids | <55 | 4 | 3 | 1.00 | 1 | 22.87 (1.19-440.93) | 0.04 | 0.02 |
|  | ≥55 | 56 | 55 | 1.00 | 1 | 0.78 (0.11-5.79) | 0.81 |  |
| Other respiratory system diseases | <55 | 23 | 23 | 1.00 | 0 | - | - | - |
|  | ≥55 | 92 | 89 | 1.00 | 3 | 1.28 (0.33-5.01) | 0.72 |  |
| Digestive diseases |  |  |  |  |  |  |  |  |
| Alcoholic liver disease | <55 | 42 | 40 | 1.00 | 2 | 10.93 (2.27-52.67) | <0.001 | 0.24 |
|  | ≥55 | 38 | 35 | 1.00 | 3 | 3.08 (0.78-12.24) | 0.11 |  |
| Other chronic liver disease | <55 | 31 | 27 | 1.00 | 4 | 11.28 (3.42-37.18) | <0.001 | 0.10 |
|  | ≥55 | 54 | 52 | 1.00 | 2 | 2.39 (0.53-10.68) | 0.26 |  |
| Urinary tract disease |  |  |  |  |  |  |  |  |
| Kidney failure | <55 | 30 | 23 | 1.00 | 7 | 24.46 (9.07-65.95) | <0.001 | 0.10 |
|  | ≥55 | 124 | 98 | 1.00 | 26 | 11.24 (6.59-19.17) | <0.001 |  |
| Self-harm, interpersonal violence |  |  |  |  |  |  |  |  |
| Suicide | <55 | 110 | 107 | 1.00 | 3 | 2.67 (0.79-9.00) | 0.11 | - |
|  | ≥55 | 37 | 37 | 1.00 | 0 | - | - |  |
| All other diseases (residual) | <55 | 211 | 201 | 1.00 | 10 | 3.92 (1.77-8.66) | 0.001 | 0.16 |
|  | ≥55 | 546 | 522 | 1.00 | 24 | 2.33 (1.45-3.75) | <0.001 |  |
| All other causes-all unknown causes | <55 | 684 | 670 | 1.00 | 14 | 1.47 (0.86-2.49) | 0.16 | 0.66 |
|  | ≥55 | 411 | 396 | 1.00 | 14 | 1.85 (1.04-3.29) | 0.04 |  |

Multivariable adjustment for age, sex, education, race, income, alcohol, smoking status, BMI, physical activity, and survey year

Supplementary Table 5. Hazard ratios and 95% confidence intervals (CIs) of all-cause mortality and cause-specific mortality among participants with chronic kidney disease vs. no chronic kidney disease, stratified by sex

|  |  | Total | No chronic kidney disease | | Chronic kidney disease | |  |  |
| --- | --- | --- | --- | --- | --- | --- | --- | --- |
|  | Sex | N  (deaths) | N  (deaths) | HR | N (deaths) | HR (95% CI) | P-value | P-value for interaction |
| All-cause mortality | Men | 4720 | 4514 | 1.00 | 206 | 2.73 (2.29-3.26) | <0.001 | 0.85 |
|  | Women | 4844 | 4608 | 1.00 | 236 | 2.64 (2.23-3.13) | <0.001 |  |
| Infections |  |  |  |  |  |  |  |  |
| Septicemia | Men | 61 | 55 | 1.00 | 6 | 4.53 (1.60-12.78) | <0.001 | 0.70 |
|  | Women | 69 | 62 | 1.00 | 7 | 6.58 (2.60-16.65) | <0.001 |  |
| Viral hepatitis | Men | 16 | 15 | 1.00 | 1 | 6.75 (0.70-65.29) | 0.1 | 0.58 |
|  | Women | 12 | 11 | 1.00 | 1 | 17.45 (2.09-145.6) | 0.01 |  |
| Human immunodeficiency virus | Men | 49 | 46 | 1.00 | 3 | 6.81 (2.52-18.42) | <0.001 | 0.63 |
|  | Women | 23 | 22 | 1.00 | 1 | 1.77 (0.29-10.88) | 0.54 |  |
| Other infectious parasitic disease | Men | 18 | 13 | 1.00 | 5 | 18.96 (5.77-62.33) | <0.001 | - |
|  | Women | 12 | 12 | 1.00 | 0 | - | - |  |
| Cancers |  |  |  |  |  |  |  |  |
| All cancers | Men | 1103 | 1082 | 1.00 | 21 | 6.81 (2.52-18.42) | <0.001 | 0.73 |
|  | Women | 1080 | 1054 | 1.00 | 26 | 1.51 (0.96-2.37) | 0.07 |  |
| Lung, trachea, bronchus | Men | 396 | 390 | 1.00 | 6 | 1.38 (0.54-3.53) | 0.5 | 0.24 |
|  | Women | 319 | 307 | 1.00 | 12 | 2.83 (1.47-5.46) | <0.001 |  |
| Kidney and renal pelvis | Men | 37 | 32 | 1.00 | 5 | 6.81 (2.52-18.42) | <0.001 | - |
|  | Women | 14 | 14 | 1.00 | 0 | - | - |  |
| Other, unspecified neoplasms | Men | 156 | 153 | 1.00 | 3 | 1.25 (0.38-4.08) | 0.71 | 0.38 |
|  | Women | 279 | 270 | 1.00 | 9 | 1.98 (0.97-4.04) | 0.06 |  |
| Endocrine, nutritional, metabolic diseases |  |  |  |  |  |  |  |  |
| Diabetes mellitus | Men | 119 | 102 | 1.00 | 17 | 8.64 (4.79-15.61) | <0.001 | 0.88 |
|  | Women | 169 | 145 | 1.00 | 24 | 8.68 (4.91-15.36) | <0.001 |  |
| Nervous system |  |  |  |  |  |  |  |  |
| Parkinson's disease | Men | 29 | 28 | 1.00 | 1 | 1.02 (0.12-8.66) | 0.99 | - |
|  | Women | 24 | 21 | 1.00 | 3 | - | - |  |
| Alzheimer's disease | Men | 46 | 46 | 1.00 | 0 | - | - | - |
|  | Women | 94 | 92 | 1.00 | 2 | 0.61 (0.13-2.85) | 0.53 |  |
| Circulatory disease |  |  |  |  |  |  |  |  |
| All circulatory diseases | Men | 1415 | 1329 | 1.00 | 86 | 3.94 (2.99-5.18) | <0.001 | 0.35 |
|  | Women | 1561 | 1475 | 1.00 | 86 | 2.91 (2.05-4.12) | <0.001 |  |
| Primary hypertension, renal disease | Men | 32 | 23 | 1.00 | 9 | 19.94 (6.05-65.80) | <0.001 | 0.25 |
|  | Women | 52 | 47 | 1.00 | 5 | 8.39 (2.70-26.08) | <0.001 |  |
| Hypertensive heart disease | Men | 54 | 51 | 1.00 | 3 | 2.86 (1.38-5.93) | 0.01 | 0.89 |
|  | Women | 55 | 52 | 1.00 | 3 | 3.19 (0.80-12.74) | 0.10 |  |
| Hypertensive heart, renal disease | Men | 4 | 2 | 1.00 | 2 | 235.88 (88.80-626.60) | <0.001 | 0.03 |
|  | Women | 10 | 9 | 1.00 | 1 | 1.80 (0.22-14.84) | 0.58 |  |
| Ischaemic heart disease | Men | 862 | 815 | 1.00 | 47 | 3.09 (2.19-4.36) | <0.001 | 0.60 |
|  | Women | 757 | 709 | 1.00 | 48 | 3.22 (1.88-5.52) | <0.001 |  |
| Acute myocardial infarction | Men | 340 | 322 | 1.00 | 18 | 3.04 (1.76-5.26) | <0.001 | 0.26 |
|  | Women | 295 | 273 | 1.00 | 22 | 4.59 (2.08-10.13) | <0.001 |  |
| Atherosclerotic cardiovascular disease | Men | 151 | 147 | 1.00 | 4 | 1.06 (0.37-3.02) | 0.92 | 0.24 |
|  | Women | 95 | 90 | 1.00 | 5 | 1.50 (0.72-3.14) | 0.28 |  |
| Other chronic ischaemic heart disease | Men | 362 | 337 | 1.00 | 25 | 4.23 (2.55-7.01) | <0.001 | 0.29 |
|  | Women | 362 | 341 | 1.00 | 21 | 2.40 (1.37-4.21) | <0.001 |  |
| Heart failure | Men | 54 | 52 | 1.00 | 2 | 2.50 (0.57-10.99) | 0.23 | 0.41 |
|  | Women | 93 | 91 | 1.00 | 2 | 0.54 (0.12-2.38) | 0.41 |  |
| All other forms of heart disease | Men | 164 | 156 | 1.00 | 8 | 3.14 (1.46-6.76) | <0.001 | 0.22 |
|  | Women | 217 | 209 | 1.00 | 8 | 1.50 (0.72-3.14) | 0.28 |  |
| Cerebrovascular disease | Men | 187 | 176 | 1.00 | 11 | 4.73 (2.23-10.05) | <0.001 | 0.12 |
|  | Women | 293 | 281 | 1.00 | 12 | 2.07 (1.03-4.18) | 0.04 |  |
| Other diseases of circulatory system | Men | 52 | 48 | 1.00 | 4 | 10.05 (2.66-37.94) | <0.001 | 0.68 |
|  | Women | 73 | 67 | 1.00 | 6 | 5.74 (2.12-15.57) | <0.001 |  |
| Aortic aneurysm and dissection | Men | 34 | 32 | 1.00 | 2 | 2.83 (0.54-14.8) | 0.22 | 0.55 |
|  | Women | 31 | 30 | 1.00 | 1 | 0.93 (0.11-7.72) | 0.94 |  |
| Other diseases of arteries or capillaries | Men | 13 | 11 | 1.00 | 2 | 40.62 (5.72-288.56) | <0.001 | <0.001 |
|  | Women | 29 | 28 | 1.00 | 1 | 0.85 (0.11-6.51) | 0.87 |  |
| Other disorders of circulatory system | Men | 5 | 5 | 1.00 | 0 | - | - | - |
|  | Women | 13 | 9 | 1.00 | 4 | 59.72 (21-169.83) | <0.001 |  |
| Respiratory diseases |  |  |  |  |  |  |  |  |
| Pneumonia | Men | 75 | 73 | 1.00 | 2 | 1.28 (0.28-5.86) | 0.75 | 0.36 |
|  | Women | 102 | 100 | 1.00 | 2 | 0.54 (0.12-2.38) | 0.41 |  |
| Other chronic lower respiratory disease | Men | 195 | 185 | 1.00 | 10 | 2.86 (1.38-5.93) | 0.01 | 0.99 |
|  | Women | 230 | 218 | 1.00 | 12 | 2.57 (1.16-5.70) | 0.02 |  |
| Pneumonitis from solids, liquids | Men | 33 | 31 | 1.00 | 2 | 2.78 (0.58-13.37) | 0.20 | - |
|  | Women | 27 | 27 | 1.00 | 0 | - | -- |  |
| Other respiratory system diseases | Men | 49 | 48 | 1.00 | 1 | 1.31 (0.17-9.80) | 0.79 | 0.59 |
|  | Women | 66 | 64 | 1.00 | 2 | 15.17 (8.72-26.39) | <0.001 |  |
| Digestive diseases |  |  |  |  |  |  |  |  |
| Alcoholic liver disease | Men | 63 | 58 | 1.00 | 5 | 7.00 (2.29-21.36) | <0.001 | - |
|  | Women | 17 | 17 | 1.00 | 0 | - | - |  |
| Other chronic liver disease | Men | 44 | 40 | 1.00 | 4 | 1.28 (0.28-5.86) | 0.75 | 0.36 |
|  | Women | 41 | 39 | 1.00 | 2 | 2.32 (0.46-11.70) | 0.31 |  |
| Urinary tract disease |  |  |  |  |  |  |  |  |
| Kidney failure | Men | 67 | 58 | 1.00 | 9 | 10.60 (4.17-26.97) | <0.001 | 0.49 |
|  | Women | 87 | 63 | 1.00 | 24 | 15.17 (8.72-26.39) | <0.001 |  |
| Self-harm, interpersonal violence |  |  |  |  |  |  |  |  |
| Suicide | Men | 117 | 115 | 1.00 | 2 | 1.70 (0.40-7.30) | 0.48 | 0.90 |
|  | Women | 30 | 29 | 1.00 | 1 | 1.98 (0.27-14.5) | 0.50 |  |
| All other diseases (residual) | Men | 321 | 307 | 1.00 | 14 | 2.66 (1.54-4.58) | <0.001 | 0.98 |
|  | Women | 436 | 416 | 1.00 | 20 | 2.52 (1.43-4.45) | 0.001 |  |
| All other causes/all unknown causes | Men | 580 | 570 | 1.00 | 10 | 1.29 (0.66-2.53) | 0.46 | 0.27 |
|  | Women | 515 | 496 | 1.00 | 19 | 1.97 (1.18-3.32) | 0.01 |  |

Multivariable adjustment for age, sex, education, race, income, alcohol, smoking status, BMI, physical activity, and survey year

Supplementary Table 6. Hazard ratios and 95% confidence intervals (CIs) of all-cause mortality and cause-specific mortality among participants with chronic kidney disease vs. no chronic kidney disease, stratified by race/ethnicity

|  |  | Total | No chronic kidney disease | | Chronic kidney disease | |  |  |
| --- | --- | --- | --- | --- | --- | --- | --- | --- |
|  | Race | N  (deaths) | N  (deaths) | HR | N (deaths) | HR (95% CI) | P-value | P-value for interaction |
| All-cause mortality | White | 6376 | 1.00 | 6139 | 237 | 2.45 (2.09-2.87) | <0.001 | 0.19 |
|  | Non-white | 3188 | 1.00 | 2983 | 205 | 3.34 (2.73-4.09) | <0.001 |  |
| Infections |  |  |  |  |  |  |  |  |
| Septicemia | White | 86 | 1.00 | 81 | 5 | 4.27 (1.50-12.14) | 0.01 | 0.41 |
|  | Non-white | 44 | 1.00 | 36 | 8 | 9.18 (3.77-22.36) | <0.001 |  |
| Viral hepatitis | White | 18 | 1.00 | 16 | 2 | 17.29 (3.67-81.50) | <0.001 | - |
|  | Non-white | 10 | 1.00 | 10 | 0 | - | - |  |
| Human immunodeficiency virus | White | 15 | 1.00 | 14 | 1 | 7.11 (0.85-59.36) | 0.07 | 0.56 |
|  | Non-white | 57 | 1.00 | 54 | 3 | 2.76 (0.77-9.82) | 0.12 |  |
| Other infectious parasitic disease | White | 19 | 1.00 | 17 | 2 | 8.69 (1.70-44.28) | 0.01 | 0.51 |
|  | Non-white | 11 | 1.00 | 8 | 3 | 12.05 (2.74-52.9) | <0.001 |  |
| Cancers |  |  |  |  |  |  |  |  |
| All cancers | White | 1550 | 1.00 | 1526 | 24 | 1.26 (0.78-2.04) | 0.34 | 0.24 |
|  | Non-white | 633 | 1.00 | 610 | 23 | 1.98 (1.19-3.28) | 0.01 |  |
| Lung, trachea, bronchus | White | 543 | 1.00 | 532 | 11 | 1.88 (0.92-3.82) | 0.08 | 0.85 |
|  | Non-white | 172 | 1.00 | 165 | 7 | 2.27 (0.88-5.86) | 0.09 |  |
| Kidney and renal pelvis | White | 35 | 1.00 | 35 | 0 | - | - | - |
|  | Non-white | 16 | 1.00 | 11 | 5 | 25.00 (7.66-81.61) | <0.001 |  |
| Other, unspecified neoplasms | White | 297 | 1.00 | 289 | 8 | 2.09 (1.02-4.28) | 0.045 | 0.60 |
|  | Non-white | 138 | 1.00 | 134 | 4 | 1.38 (0.43-4.40) | 0.59 |  |
| Endocrine, nutritional, metabolic diseases |  |  |  |  |  |  |  |  |
| Diabetes mellitus | White | 162 | 1.00 | 145 | 17 | 5.91 (3.24-10.80) | <0.001 | 0.06 |
|  | Non-white | 126 | 1.00 | 102 | 24 | 11.83 (6.55-21.38) | <0.001 |  |
| Nervous system |  |  |  |  |  |  |  |  |
| Parkinson's disease | White | 45 | 1.00 | 41 | 4 | 5.98 (2.10-17.08) | <0.001 | - |
|  | Non-white | 8 | 1.00 | 8 | 0 | - | - |  |
| Alzheimer's disease | White | 115 | 1.00 | 114 | 1 | 0.35 (0.05-2.58) | 0.30 | - |
|  | Non-white | 25 | 1.00 | 24 | 1 | 0.81 (0.09-7.08) | 0.85 |  |
| Circulatory disease |  |  |  |  |  |  |  |  |
| All circulatory diseases | White | 2063 | 1.00 | 1973 | 90 | 2.82 (2.18-3.65) | <0.001 | 0.05 |
|  | Non-white | 913 | 1.00 | 831 | 82 | 4.63 (3.24-6.61) | <0.001 |  |
| Primary hypertension, renal disease | White | 55 | 1.00 | 50 | 5 | 7.00 (2.35-20.85) | <0.001 | 0.12 |
|  | Non-white | 29 | 1.00 | 20 | 9 | 36.59 (11.07-120.99) | <0.001 |  |
| Hypertensive heart disease | White | 59 | 1.00 | 55 | 4 | 5.34 (1.68-16.95) | 0.01 | 0.12 |
|  | Non-white | 50 | 1.00 | 48 | 2 | 1.25 (0.23-6.75) | 0.79 |  |
| Hypertensive heart, renal disease | White | 7 | 1.00 | 7 | 0 | - | - | - |
|  | Non-white | 7 | 1.00 | 4 | 3 | 49.91 (3.51-710.41) | <0.001 |  |
| Ischaemic heart disease | White | 1111 | 1.00 | 1063 | 48 | 2.58 (1.86-3.59) | <0.001 | 0.11 |
|  | Non-white | 508 | 1.00 | 461 | 47 | 4.4 (2.57-7.53) | <0.001 |  |
| Acute myocardial infarction | White | 457 | 1.00 | 437 | 20 | 2.68 (1.62-4.44) | <0.001 | 0.08 |
|  | Non-white | 178 | 1.00 | 158 | 20 | 6.55 (2.56-16.74) | <0.001 |  |
| Atherosclerotic cardiovascular disease | White | 145 | 1.00 | 143 | 2 | 0.49 (0.12-1.99) | 0.32 | 0.04 |
|  | Non-white | 101 | 1.00 | 94 | 7 | 3.13 (1.34-7.31) | 0.01 |  |
| Other chronic ischaemic heart disease | White | 498 | 1.00 | 472 | 26 | 3.17 (1.99-5.06) | <0.001 | 0.89 |
|  | Non-white | 226 | 1.00 | 206 | 20 | 3.31 (1.89-5.81) | <0.001 |  |
| Heart failure | White | 108 | 1.00 | 105 | 3 | 1.54 (0.45-5.31) | 0.50 | 0.97 |
|  | Non-white | 39 | 1.00 | 38 | 1 | 1.74 (0.22-13.80) | 0.60 |  |
| All other forms of heart disease | White | 277 | 1.00 | 266 | 11 | 2.19 (1.15-4.17) | 0.02 | 0.79 |
|  | Non-white | 104 | 1.00 | 99 | 5 | 1.95 (0.74-5.15) | 0.18 |  |
| Cerebrovascular disease | White | 332 | 1.00 | 322 | 10 | 2.20 (1.06-4.56) | 0.03 | 0.14 |
|  | Non-white | 148 | 1.00 | 135 | 13 | 4.86 (2.35-10.04) | <0.001 |  |
| Other diseases of circulatory system | White | 100 | 1.00 | 92 | 8 | 7.30 (2.79-19.09) | <0.001 | 0.85 |
|  | Non-white | 25 | 1.00 | 23 | 2 | 10.97 (2.00-60.04) | 0.01 |  |
| Aortic aneurysm and dissection | White | 53 | 1.00 | 51 | 2 | 2.05 (0.44-9.50) | 0.36 | 0.64 |
|  | Non-white | 12 | 1.00 | 11 | 1 | 2.85 (0.35-23.11) | 0.33 |  |
| Other diseases of arteries or capillaries | White | 36 | 1.00 | 33 | 3 | 11.64 (2.30-58.97) | <0.001 | - |
|  | Non-white | 6 | 1.00 | 6 | 0 | - | - |  |
| Other disorders of circulatory system | White | 11 | 1.00 | 8 | 3 | - | - | - |
|  | Non-white | 7 | 1.00 | 6 | 1 | 47.16 (9.49-234.39) | <0.001 |  |
| Respiratory diseases |  |  |  |  |  |  |  |  |
| Pneumonia | White | 121 | 1.00 | 119 | 2 | 0.74 (0.18-3.08) | 0.67 | 0.66 |
|  | Non-white | 56 | 1.00 | 54 | 2 | 1.31 (0.24-7.07) | 0.76 |  |
| Other chronic lower respiratory disease | White | 371 | 1.00 | 351 | 20 | 3.05 (1.76-5.29) | <0.001 | 0.03 |
|  | Non-white | 54 | 1.00 | 52 | 2 | 0.67 (0.15-3.07) | 0.60 |  |
| Pneumonitis from solids, liquids | White | 50 | 1.00 | 48 | 2 | 2.22 (0.50-9.82) | 0.29 | - |
|  | Non-white | 10 | 1.00 | 10 | 0 | - | - |  |
| Other respiratory system diseases | White | 80 | 1.00 | 78 | 2 | 1.29 (0.29-5.82) | 0.74 | 0.30 |
|  | Non-white | 35 | 1.00 | 34 | 1 | 0.38 (0.05-2.90) | 0.35 |  |
| Digestive diseases |  |  |  |  |  |  |  |  |
| Alcoholic liver disease | White | 44 | 1.00 | 42 | 2 | 3.33 (0.73-15.29) | 0.12 | 0.41 |
|  | Non-white | 36 | 1.00 | 33 | 3 | 9.86 (2.17-44.73) | <0.001 |  |
| Other chronic liver disease | White | 63 | 1.00 | 59 | 4 | 4.11 (1.32-12.79) | 0.02 | 0.91 |
|  | Non-white | 22 | 1.00 | 20 | 2 | 5.55 (1.19-25.94) | 0.03 |  |
| Urinary tract disease |  |  |  |  |  |  |  |  |
| Kidney failure | White | 97 | 1.00 | 81 | 16 | 12.40 (6.59-23.34) | <0.001 | 0.70 |
|  | Non-white | 57 | 1.00 | 40 | 17 | 14.24 (6.99-29.05) | <0.001 |  |
| Self-harm, interpersonal violence |  |  |  |  |  |  |  |  |
| Suicide | White | 116 | 1.00 | 113 | 3 | 2.38 (0.68-8.36) | 0.18 | - |
|  | Non-white | 31 | 1.00 | 31 | 0 | - | - |  |
| All other diseases (residual) | White | 521 | 1.00 | 500 | 21 | 2.60 (1.55-4.37) | <0.001 | 0.87 |
|  | Non-white | 236 | 1.00 | 223 | 13 | 2.72 (1.50-4.93) | 0.001 |  |
| All other causes/all unknown causes | White | 429 | 1.00 | 417 | 12 | 1.49 (0.79-2.81) | 0.22 | 0.75 |
|  | Non-white | 666 | 1.00 | 649 | 17 | 1.83 (1.05-3.20) | 0.03 |  |

Multivariable adjustment for age, sex, education, race, income, alcohol, smoking status, BMI, physical activity, and survey year

Supplementary Table 7. Hazard ratios and 95% confidence intervals (CIs) of all-cause mortality and cause-specific mortality among participants with chronic kidney disease vs. no chronic kidney disease, stratified by BMI

|  |  | Total | No chronic kidney disease | | Chronic kidney disease | |  |  |
| --- | --- | --- | --- | --- | --- | --- | --- | --- |
|  | BMI | N  (deaths) | N  (deaths) | HR | N (deaths) | HR (95% CI) | P-value | P-value for interaction |
| All-cause mortality | <25 | 4057 | 3871 | 1.00 | 186 | 2.66 (2.23-3.18) | <0.001 | 0.73 |
|  | 25-<30 | 3136 | 3009 | 1.00 | 127 | 2.91 (2.28-3.70) | <0.001 |  |
|  | ≥30 | 2021 | 1904 | 1.00 | 117 | 2.51 (1.96-3.21) | <0.001 |  |
| Infections |  |  |  |  |  |  |  |  |
| Septicemia | <25 | 52 | 46 | 1.00 | 6 | 4.95 (1.79-13.70) | <0.001 | <0.001 |
|  | 25-<30 | 37 | 36 | 1.00 | 1 | 2.40 (0.33-17.40) | 0.38 |  |
|  | ≥30 | 33 | 27 | 1.00 | 6 | 9.12 (2.63-31.69) | <0.001 |  |
| Viral hepatitis | <25 | 13 | 11 | 1.00 | 2 | 30.84 (5.76-165.09) | <0.001 | - |
|  | 25-<30 | 9 | 9 | 1.00 | 0 | - | - |  |
|  | ≥30 | 4 | 4 | 1.00 | 0 | - | - |  |
| Human immunodeficiency virus | <25 | 42 | 40 | 1.00 | 2 | 2.85 (0.59-13.87) | 0.19 | <0.001 |
|  | 25-<30 | 20 | 18 | 1.00 | 2 | 6.66 (1.22-36.48) | 0.03 |  |
|  | ≥30 | 10 | 10 | 1.00 | 0 | - | - |  |
| Other infectious parasitic disease | <25 | 14 | 13 | 1.00 | 1 | 6.42 (1.18-35.00) | 0.03 | <0.001 |
|  | 25-<30 | 10 | 7 | 1.00 | 3 | 19.30 (2.82-131.92) | <0.001 |  |
|  | ≥30 | 4 | 3 | 1.00 | 1 | - | - |  |
| Cancers |  |  |  |  |  |  |  |  |
| All cancers | <25 | 922 | 906 | 1.00 | 16 | 1.28 (0.74-2.21) | 0.38 | 0.07 |
|  | 25-<30 | 782 | 762 | 1.00 | 20 | 2.40 (1.43-4.01) | <0.001 |  |
|  | ≥30 | 411 | 401 | 1.00 | 10 | 0.73 (0.37-1.44) | 0.37 |  |
| Lung, trachea, bronchus | <25 | 368 | 364 | 1.00 | 4 | 0.68 (0.24-1.94) | 0.47 | <0.001 |
|  | 25-<30 | 234 | 224 | 1.00 | 10 | 4.63 (2.26-9.48) | <0.001 |  |
|  | ≥30 | 92 | 88 | 1.00 | 4 | 1.48 (0.51-4.33) | 0.47 |  |
| Kidney and renal pelvis | <25 | 14 | 13 | 1.00 | 1 | - | - | 0.06 |
|  | 25-<30 | 19 | 17 | 1.00 | 2 | 4.37 (1.02-18.74) | 0.047 |  |
|  | ≥30 | 18 | 16 | 1.00 | 2 | 4.86 (0.99-23.79) | 0.05 |  |
| Other, unspecified neoplasms | <25 | 173 | 167 | 1.00 | 6 | 2.48 (1.04-5.89) | 0.04 | 0.54 |
|  | 25-<30 | 156 | 153 | 1.00 | 3 | 1.88 (0.58-6.12) | 0.30 |  |
|  | ≥30 | 91 | 89 | 1.00 | 2 | 0.68 (0.13-3.47) | 0.65 |  |
| Endocrine, nutritional, metabolic diseases |  |  |  |  |  |  |  |  |
| Diabetes mellitus | <25 | 82 | 69 | 1.00 | 13 | 11.76 (5.89-23.51) | <0.001 | 0.17 |
|  | 25-<30 | 95 | 84 | 1.00 | 11 | 4.95 (2.25-10.93) | <0.001 |  |
|  | ≥30 | 96 | 81 | 1.00 | 15 | 7.54 (3.81-14.92) | <0.001 |  |
| Nervous system |  |  |  |  |  |  |  |  |
| Parkinson's disease | <25 | 34 | 31 | 1.00 | 3 | 5.62 (1.84-17.22) | <0.001 | 0.79 |
|  | 25-<30 | 13 | 12 | 1.00 | 1 | 5.99 (0.73-49.25) | 0.10 |  |
|  | ≥30 | 5 | 5 | 1.00 | 0 | - | - |  |
| Alzheimer's disease | <25 | 83 | 82 | 1.00 | 1 | 0.49 (0.07-3.69) | 0.49 | 0.89 |
|  | 25-<30 | 34 | 33 | 1.00 | 1 | 0.69 (0.09-5.17) | 0.72 |  |
|  | ≥30 | 17 | 17 | 1.00 | 0 | - | - |  |
| Circulatory disease |  |  |  |  |  |  |  |  |
| All circulatory diseases | <25 | 1219 | 1147 | 1.00 | 72 | 3.27 (2.37-4.52) | <0.001 | 0.61 |
|  | 25-<30 | 962 | 915 | 1.00 | 47 | 3.97 (2.56-6.15) | <0.001 |  |
|  | ≥30 | 709 | 659 | 1.00 | 50 | 3.11 (2.14-4.54) | <0.001 |  |
| Primary hypertension, renal disease | <25 | 37 | 30 | 1.00 | 7 | 14.65 (5.13-41.86) | <0.001 | 0.64 |
|  | 25-<30 | 21 | 19 | 1.00 | 2 | 6.65 (1.32-33.41) | 0.02 |  |
|  | ≥30 | 24 | 19 | 1.00 | 5 | 25.74 (5.22-126.96) | <0.001 |  |
| Hypertensive heart disease | <25 | 32 | 31 | 1.00 | 1 | 1.56 (0.19-12.74) | 0.68 | <0.001 |
|  | 25-<30 | 33 | 31 | 1.00 | 2 | 3.16 (0.63-15.91) | 0.16 |  |
|  | ≥30 | 40 | 37 | 1.00 | 3 | 4.59 (1.04-20.33) | 0.05 |  |
| Hypertensive heart, renal disease | <25 | 3 | 2 | 1.00 | 1 | 18.79 (1.22-288.32) | 0.04 | 0.91 |
|  | 25-<30 | 2 | 2 | 1.00 | 0 | - | - |  |
|  | ≥30 | 7 | 5 | 1.00 | 2 | 24.67 (3.57-170.58) | <0.001 |  |
| Ischaemic heart disease | <25 | 647 | 609 | 1.00 | 38 | 2.66 (1.80-3.93) | <0.001 | <0.001 |
|  | 25-<30 | 538 | 515 | 1.00 | 23 | 3.70 (1.84-7.48) | <0.001 |  |
|  | ≥30 | 388 | 354 | 1.00 | 34 | 3.68 (2.34-5.79) | <0.001 |  |
| Acute myocardial infarction | <25 | 242 | 228 | 1.00 | 14 | 2.61 (1.41-4.84) | <0.001 | <0.001 |
|  | 25-<30 | 219 | 204 | 1.00 | 15 | 7.15 (2.99-17.13) | <0.001 |  |
|  | ≥30 | 157 | 146 | 1.00 | 11 | 2.55 (1.28-5.10) | 0.01 |  |
| Atherosclerotic cardiovascular disease | <25 | 99 | 97 | 1.00 | 2 | 0.84 (0.20-3.57) | 0.82 | 0.07 |
|  | 25-<30 | 76 | 75 | 1.00 | 1 | 0.50 (0.07-3.57) | 0.49 |  |
|  | ≥30 | 66 | 60 | 1.00 | 6 | 4.63 (1.79-11.99) | 0.00 |  |
| Other chronic ischaemic heart disease | <25 | 301 | 279 | 1.00 | 22 | 3.55 (2.08-6.05) | 0.00 | 0.27 |
|  | 25-<30 | 236 | 229 | 1.00 | 7 | 1.89 (0.79-4.50) | 0.15 |  |
|  | ≥30 | 163 | 146 | 1.00 | 17 | 4.56 (2.34-8.89) | <0.001 |  |
| Heart failure | <25 | 59 | 56 | 1.00 | 3 | 2.75 (0.72-10.48) | 0.14 | 0.77 |
|  | 25-<30 | 50 | 49 | 1.00 | 1 | 1.58 (0.23-10.57) | 0.64 |  |
|  | ≥30 | 34 | 34 | 1.00 | 0 | - | - |  |
| All other forms of heart disease | <25 | 149 | 143 | 1.00 | 6 | 1.97 (0.85-4.55) | 0.11 | 0.63 |
|  | 25-<30 | 117 | 112 | 1.00 | 5 | 3.00 (1.14-7.90) | 0.03 |  |
|  | ≥30 | 106 | 102 | 1.00 | 4 | 1.55 (0.47-5.14) | 0.47 |  |
| Cerebrovascular disease | <25 | 225 | 214 | 1.00 | 11 | 3.39 (1.58-7.26) | <0.001 | 0.01 |
|  | 25-<30 | 159 | 149 | 1.00 | 10 | 4.72 (2.19-10.19) | <0.001 |  |
|  | ≥30 | 84 | 83 | 1.00 | 1 | 0.15 (0.02-1.08) | 0.06 |  |
| Other diseases of circulatory system | <25 | 57 | 52 | 1.00 | 5 | 8.91 (3.10-25.63) | <0.001 | 0.85 |
|  | 25-<30 | 38 | 34 | 1.00 | 4 | 11.71 (2.69-51.05) | <0.001 |  |
|  | ≥30 | 24 | 24 | 1.00 | 0 | - | - |  |
| Aortic aneurysm and dissection | <25 | 29 | 28 | 1.00 | 1 | 1.15 (0.14-9.36) | 0.90 | 0.31 |
|  | 25-<30 | 21 | 19 | 1.00 | 2 | 4.66 (0.85-25.54) | 0.08 |  |
|  | ≥30 | 13 | 13 | 1.00 | 0 | - | - |  |
| Other diseases of arteries or capillaries | <25 | 17 | 16 | 1.00 | 1 | 6.94 (0.80-60.26) | 0.08 | <0.001 |
|  | 25-<30 | 12 | 11 | 1.00 | 1 | 26.59 (2.13-331.50) | 0.01 |  |
|  | ≥30 | 9 | 9 | 1.00 | 0 | - | - |  |
| Other disorders of circulatory system | <25 | 11 | 8 | 1.00 | 3 | 48.86 (16.38-145.79) | <0.001 | 0.35 |
|  | 25-<30 | 5 | 4 | 1.00 | 1 | 30.92 (2.93-326.12) | <0.001 |  |
|  | ≥30 | 2 | 2 | 1.00 | 0 | - | - |  |
| Respiratory diseases |  |  |  |  |  |  |  |  |
| Pneumonia | <25 | 85 | 85 | 1.00 | 0 | - | - | <0.001 |
|  | 25-<30 | 50 | 47 | 1.00 | 3 | 3.29 (0.89-12.20) | 0.07 |  |
|  | ≥30 | 27 | 26 | 1.00 | 1 | 0.37 (0.05-2.96) | 0.35 |  |
| Other chronic lower respiratory disease | <25 | 244 | 231 | 1.00 | 13 | 2.15 (1.12-4.14) | 0.02 | 0.54 |
|  | 25-<30 | 105 | 100 | 1.00 | 5 | 3.90 (1.51-10.11) | 0.01 |  |
|  | ≥30 | 62 | 59 | 1.00 | 3 | 2.11 (0.34-12.99) | 0.42 |  |
| Pneumonitis from solids, liquids | <25 | 27 | 26 | 1.00 | 1 | 1.87 (0.25-14.15) | 0.54 | <0.001 |
|  | 25-<30 | 18 | 18 | 1.00 | 0 | - | - |  |
|  | ≥30 | 10 | 10 | 1.00 | 0 | - | - |  |
| Other respiratory system diseases | <25 | 48 | 46 | 1.00 | 2 | 2.03 (0.38-10.81) | 0.41 | 0.57 |
|  | 25-<30 | 39 | 38 | 1.00 | 1 | 0.86 (0.11-6.59) | 0.88 |  |
|  | ≥30 | 25 | 25 | 1.00 | 0 | - | - |  |
| Digestive diseases |  |  |  |  |  |  |  |  |
| Alcoholic liver disease | <25 | 33 | 31 | 1.00 | 2 | 3.02 (0.56-16.25) | 0.20 | <0.001 |
|  | 25-<30 | 29 | 27 | 1.00 | 2 | 12.13 (1.86-79.34) | 0.01 |  |
|  | ≥30 | 16 | 15 | 1.00 | 1 | 4.98 (0.69-35.98) | 0.11 |  |
| Other chronic liver disease | <25 | 34 | 32 | 1.00 | 2 | 3.66 (0.74-18.2) | 0.11 | 0.32 |
|  | 25-<30 | 26 | 24 | 1.00 | 2 | 6.99 (1.39-35.23) | 0.02 |  |
|  | ≥30 | 21 | 20 | 1.00 | 1 | 1.64(0.19-14.19) | 0.65 |  |
| Urinary tract disease |  |  |  |  |  |  |  |  |
| Kidney failure | <25 | 63 | 48 | 1.00 | 15 | 15.59 (8.14-29.86) | <0.001 | 0.02 |
|  | 25-<30 | 43 | 37 | 1.00 | 6 | 3.33 (1.25-8.84) | 0.02 |  |
|  | ≥30 | 45 | 33 | 1.00 | 12 | 24.02 (9.21-62.63) | <0.001 |  |
| Self-harm, interpersonal violence |  |  |  |  |  |  |  |  |
| Suicide | <25 | 67 | 65 | 1.00 | 2 | 2.11 (0.50-8.95) | 0.31 | <0.001 |
|  | 25-<30 | 47 | 46 | 1.00 | 1 | 2.77 (0.33-23.40) | 0.35 |  |
|  | ≥30 | 31 | 31 | 1.00 | 0 | - | - |  |
| All other diseases (residual) | <25 | 306 | 290 | 1.00 | 16 | 3.73 (2.16-6.47) | <0.001 | 0.20 |
|  | 25-<30 | 240 | 232 | 1.00 | 8 | 1.73 (0.75-3.97) | 0.20 |  |
|  | ≥30 | 173 | 165 | 1.00 | 8 | 1.86 (0.97-3.59) | 0.06 |  |
| All other causes/all unknown causes | <25 | 455 | 443 | 1.00 | 12 | 1.64 (0.86-3.10) | 0.13 | 0.67 |
|  | 25-<30 | 373 | 364 | 1.00 | 9 | 1.71 (0.79-3.71) | 0.17 |  |
|  | ≥30 | 208 | 201 | 1.00 | 7 | 1.66 (0.71-3.87) | 0.24 |  |

Multivariable adjustment for age, sex, education, race, income, alcohol, smoking status, BMI, physical activity, and survey year

Supplementary Table 8. Hazard ratios and 95% confidence intervals (CIs) of all-cause mortality and cause-specific mortality among participants with chronic kidney disease vs. no chronic kidney disease, stratified by physical activity

|  |  | Total | No chronic kidney disease | | Chronic kidney disease | |  |  |
| --- | --- | --- | --- | --- | --- | --- | --- | --- |
|  | Physical activity | N  (deaths) | N  (deaths) | HR | N (deaths) | HR (95% CI) | P-value | P-value for interaction |
| All-cause mortality | Inactive | 5771 | 5436 | 1.00 | 335 | 2.60 (2.26-3.00) | <0.001 | 0.19 |
|  | Insufficient | 1473 | 1423 | 1.00 | 50 | 2.74 (1.96-3.83) | <0.001 |  |
|  | Sufficient | 2067 | 2025 | 1.00 | 42 | 2.64 (1.79-3.88) | <0.001 |  |
| Infections |  |  |  |  |  |  |  |  |
| Septicemia | Inactive | 83 | 72 | 1.00 | 11 | 5.72 (2.80-11.66) | <0.001 | <0.001 |
|  | Insufficient | 21 | 20 | 1.00 | 1 | 8.95 (0.94-84.91) | 0.06 |  |
|  | Sufficient | 25 | 24 | 1.00 | 1 | 3.47 (0.41-29.65) | 0.26 |  |
| Viral hepatitis | Inactive | 14 | 13 | 1.00 | 1 | 11.04 (1.60-76.15) | 0.02 | 0.52 |
|  | Insufficient | 4 | 4 | 1.00 | 0 | - | - |  |
|  | Sufficient | 8 | 7 | 1.00 | 1 | 28.04 (2.40-327.54) | 0.01 |  |
| Human immunodeficiency virus | Inactive | 44 | 41 | 1.00 | 3 | 3.22 (0.86-11.99) | 0.08 | <0.001 |
|  | Insufficient | 14 | 13 | 1.00 | 1 | - | - |  |
|  | Sufficient | 13 | 13 | 1.00 | 0 | - | - |  |
| Other infectious parasitic disease | Inactive | 19 | 14 | 1.00 | 5 | 18.50 (5.52-61.98) | <0.001 | - |
|  | Insufficient | 5 | 5 | 1.00 | 0 | - | - |  |
|  | Sufficient | 6 | 6 | 1.00 | 0 | - | - |  |
| Cancers |  |  |  |  |  |  |  |  |
| All cancers | Inactive | 1172 | 1137 | 1.00 | 35 | 1.50 (1.00-2.24) | 0.05 | 0.84 |
|  | Insufficient | 371 | 364 | 1.00 | 7 | 1.76 (0.77-4.02) | 0.18 |  |
|  | Sufficient | 584 | 580 | 1.00 | 4 | 1.00 (0.37-2.73) | 1.00 |  |
| Lung, trachea, bronchus | Inactive | 421 | 408 | 1.00 | 13 | 1.74 (0.88-3.47) | 0.11 | 0.36 |
|  | Insufficient | 101 | 98 | 1.00 | 3 | 3.49 (1.06-11.42) | 0.04 |  |
|  | Sufficient | 178 | 177 | 1.00 | 1 | 1.19 (0.19-7.51) | 0.86 |  |
| Kidney and renal pelvis | Inactive | 28 | 25 | 1.00 | 3 | 3.55 (0.97-13.00) | 0.06 | <0.001 |
|  | Insufficient | 14 | 12 | 1.00 | 2 | 10.67 (2.22-51.28) | <0.001 |  |
|  | Sufficient | 8 | 8 | 1.00 | 0 | - | - |  |
| Other, unspecified neoplasms | Inactive | 219 | 209 | 1.00 | 10 | 1.99 (1.01-3.93) | 0.048 | 0.78 |
|  | Insufficient | 92 | 92 | 1.00 | 0 | - | - |  |
|  | Sufficient | 119 | 117 | 1.00 | 2 | 2.41 (0.58-10.04) | 0.23 |  |
| Endocrine, nutritional, metabolic diseases |  |  |  |  |  |  |  |  |
| Diabetes mellitus | Inactive | 189 | 155 | 1.00 | 34 | 9.62 (6.01-15.41) | <0.001 | <0.001 |
|  | Insufficient | 40 | 37 | 1.00 | 3 | 3.25 (0.86-12.27) | 0.08 |  |
|  | Sufficient | 52 | 49 | 1.00 | 3 | 10.58 (3.35-33.37) | <0.001 |  |
| Nervous system |  |  |  |  |  |  |  |  |
| Parkinson's disease | Inactive | 29 | 27 | 1.00 | 2 | 3.27 (0.84-12.67) | 0.09 | 0.03 |
|  | Insufficient | 11 | 11 | 1.00 | 0 | - | - |  |
|  | Sufficient | 12 | 10 | 1.00 | 2 | 51.38 (10.95-241.02) | <0.001 |  |
| Alzheimer's disease | Inactive | 85 | 84 | 1.00 | 1 | 0.36 (0.05-2.74) | 0.33 | 0.33 |
|  | Insufficient | 26 | 26 | 1.00 | 0 | - | - |  |
|  | Sufficient | 27 | 26 | 1.00 | 1 | 1.62 (0.19-13.63) | 0.66 |  |
| Circulatory disease |  |  |  |  |  |  |  |  |
| All circulatory diseases | Inactive | 1894 | 1763 | 1.00 | 131 | 3.06 (2.38-3.93) | <0.001 | 0.03 |
|  | Insufficient | 461 | 443 | 1.00 | 18 | 3.30 (1.78-6.14) | <0.001 |  |
|  | Sufficient | 542 | 529 | 1.00 | 13 | 4.10 (1.96-8.56) | <0.001 |  |
| Primary hypertension, renal disease | Inactive | 63 | 52 | 1.00 | 11 | 10.17 (4.76-21.74) | <0.001 | 0.06 |
|  | Insufficient | 10 | 9 | 1.00 | 1 | 30.42 (3.05-303.63) | <0.001 |  |
|  | Sufficient | 8 | 8 | 1.00 | 0 | - | - |  |
| Hypertensive heart disease | Inactive | 62 | 59 | 1.00 | 3 | 2.49 (0.64-9.69) | 0.19 | <0.001 |
|  | Insufficient | 19 | 18 | 1.00 | 1 | 0.89 (0.11-7.23) | 0.91 |  |
|  | Sufficient | 23 | 21 | 1.00 | 2 | 11.92 (2.51-56.62) | <0.001 |  |
| Hypertensive heart, renal disease | Inactive | 10 | 8 | 1.00 | 2 | 7.04 (1.36-36.42) | 0.02 | 0.02 |
|  | Insufficient | 1 | 1 | 1.00 | 0 | - | - |  |
|  | Sufficient | 2 | 1 | 1.00 | 1 | - | - |  |
| Ischaemic heart disease | Inactive | 1032 | 958 | 1.00 | 74 | 3.17 (2.20-4.58) | <0.001 | 0.07 |
|  | Insufficient | 251 | 241 | 1.00 | 10 | 2.40 (1.10-5.27) | 0.03 |  |
|  | Sufficient | 296 | 291 | 1.00 | 5 | 2.31 (0.79-6.75) | 0.13 |  |
| Acute myocardial infarction | Inactive | 415 | 381 | 1.00 | 34 | 4.300 (2.33-7.93) | <0.001 | 0.03 |
|  | Insufficient | 92 | 89 | 1.00 | 3 | 1.57 (0.42-5.82) | 0.50 |  |
|  | Sufficient | 113 | 112 | 1.00 | 1 | 0.61 (0.08-4.50) | 0.63 |  |
| Atherosclerotic cardiovascular disease | Inactive | 145 | 137 | 1.00 | 8 | 1.69 (0.79-3.60) | 0.17 | - |
|  | Insufficient | 44 | 43 | 1.00 | 1 | 1.86 (0.24-14.67) | 0.56 |  |
|  | Sufficient | 50 | 50 | 1.00 | 0 | - | 0.01 |  |
| Other chronic ischaemic heart disease | Inactive | 466 | 434 | 1.00 | 32 | 2.72 (1.76-4.20) | <0.001 | 0.04 |
|  | Insufficient | 114 | 108 | 1.00 | 6 | 3.42 (1.25-9.36) | 0.02 |  |
|  | Sufficient | 127 | 123 | 1.00 | 4 | 4.76 (1.38-16.45) | 0.01 |  |
| Heart failure | Inactive | 104 | 102 | 1.00 | 2 | 1.02 (0.23-4.46) | 0.98 | 0.26 |
|  | Insufficient | 23 | 22 | 1.00 | 1 | 2.93 (0.34-25.70) | 0.33 |  |
|  | Sufficient | 19 | 18 | 1.00 | 1 | 7.61 (0.74-77.75) | 0.09 |  |
| All other forms of heart disease | Inactive | 229 | 214 | 1.00 | 15 | 2.48 (1.44-4.25) | <0.001 | 0.15 |
|  | Insufficient | 66 | 66 | 1.00 | 0 | - | - |  |
|  | Sufficient | 75 | 75 | 1.00 | 0 | - | 0.01 |  |
| Cerebrovascular disease | Inactive | 299 | 282 | 1.00 | 17 | 2.23 (1.24-3.99) | 0.01 | 0.12 |
|  | Insufficient | 72 | 69 | 1.00 | 3 | 8.41 (2.60-27.15) | <0.001 |  |
|  | Sufficient | 96 | 93 | 1.00 | 3 | 4.80 (1.28-17.96) | 0.02 |  |
| Other diseases of circulatory system | Inactive | 83 | 76 | 1.00 | 7 | 4.94 (2.07-11.82) | <0.001 | 0.06 |
|  | Insufficient | 17 | 16 | 1.00 | 1 | 1.56 (0.20-11.98) | 0.67 |  |
|  | Sufficient | 21 | 20 | 1.00 | 1 | 22.65 (2.43-211.52) | 0.01 |  |
| Aortic aneurysm and dissection | Inactive | 42 | 40 | 1.00 | 2 | 2.01 (0.42-9.53) | 0.38 | <0.001 |
|  | Insufficient | 11 | 10 | 1.00 | 1 | - | - |  |
|  | Sufficient | 11 | 11 | 1.00 | 0 | - | - |  |
| Other diseases of arteries or capillaries | Inactive | 32 | 31 | 1.00 | 1 | 0.65 (0.08-5.03) | 0.68 | <0.001 |
|  | Insufficient | 3 | 3 | 1.00 | 0 | - | - |  |
|  | Sufficient | 6 | 5 | 1.00 | 1 | 72.40 (4.59-1142.58) | <0.001 |  |
| Other disorders of circulatory system | Inactive | 9 | 5 | 1.00 | 4 | 77.21 (21.94-271.72) | <0.001 | - |
|  | Insufficient | 3 | 3 | 1.00 | 0 | - | - |  |
|  | Sufficient | 4 | 4 | 1.00 | 0 | - | - |  |
| Respiratory diseases |  |  |  |  |  |  |  |  |
| Pneumonia | Inactive | 125 | 121 | 1.00 | 4 | 1.15 (0.37-3.51) | 0.81 | <0.001 |
|  | Insufficient | 16 | 16 | 1.00 | 0 | - | - |  |
|  | Sufficient | 26 | 26 | 1.00 | 0 | - | - |  |
| Other chronic lower respiratory disease | Inactive | 314 | 296 | 1.00 | 18 | 2.61 (1.45-4.70) | <0.001 | 0.17 |
|  | Insufficient | 46 | 43 | 1.00 | 3 | 6.18 (1.63-23.38) | 0.01 |  |
|  | Sufficient | 55 | 55 | 1.00 | 0 | - | - |  |
| Pneumonitis from solids, liquids | Inactive | 38 | 37 | 1.00 | 1 | 1.11 (0.14-8.82) | 0.92 | <0.001 |
|  | Insufficient | 14 | 13 | 1.00 | 1 | 2.90 (0.34-24.97) | 0.33 |  |
|  | Sufficient | 6 | 6 | 1.00 | 0 | - | - |  |
| Other respiratory system diseases | Inactive | 77 | 74 | 1.00 | 3 | 1.36 (0.35-5.33) | 0.66 | <0.001 |
|  | Insufficient | 17 | 17 | 1.00 | 0 | - | - |  |
|  | Sufficient | 13 | 13 | 1.00 | 0 | - | - |  |
| Digestive diseases |  |  |  |  |  |  |  |  |
| Alcoholic liver disease | Inactive | 49 | 44 | 1.00 | 5 | 7.04 (2.13-23.19) | <0.001 | <0.001 |
|  | Insufficient | 15 | 15 | 1.00 | 0 | - | - |  |
|  | Sufficient | 13 | 13 | 1.00 | 0 | - | - |  |
| Other chronic liver disease | Inactive | 58 | 53 | 1.00 | 5 | 4.61 (1.68-12.69) | 0.00 | 0.58 |
|  | Insufficient | 11 | 10 | 1.00 | 1 | 5.67 (0.37-87.52) | 0.21 |  |
|  | Sufficient | 15 | 15 | 1.00 | 0 | - | - |  |
| Urinary tract disease |  |  |  |  |  |  |  |  |
| Kidney failure | Inactive | 114 | 87 | 1.00 | 27 | 12.74 (7.5-21.65) | <0.001 | <0.001 |
|  | Insufficient | 17 | 14 | 1.00 | 3 | 16.69 (4.09-68.07) | <0.001 |  |
|  | Sufficient | 18 | 15 | 1.00 | 3 | 32.96 (9.68-112.16) | <0.001 |  |
| Self-harm, interpersonal violence |  |  |  |  |  |  |  |  |
| Suicide | Inactive | 67 | 66 | 1.00 | 1 | 0.72 (0.10-5.20) | 0.74 | <0.001 |
|  | Insufficient | 22 | 21 | 1.00 | 1 | 5.81 (0.69-48.83) | 0.11 |  |
|  | Sufficient | 53 | 52 | 1.00 | 1 | 2.23 (0.30-16.36) | 0.43 |  |
| All other diseases (residual) | Inactive | 478 | 452 | 1.00 | 26 | 2.31 (1.45-3.70) | 0.001 | 0.18 |
|  | Insufficient | 122 | 118 | 1.00 | 4 | 3.25 (1.09-9.69) | 0.04 |  |
|  | Sufficient | 135 | 133 | 1.00 | 2 | 1.67 (0.41-6.82) | 0.47 |  |
| All other causes/all unknown causes | Inactive | 607 | 589 | 1.00 | 18 | 1.35 (0.79-2.31) | 0.28 | <0.001 |
|  | Insufficient | 166 | 161 | 1.00 | 5 | 2.77 (1.11-6.93) | 0.03 |  |
|  | Sufficient | 297 | 291 | 1.00 | 6 | 2.24 (0.86-5.89) | 0.10 |  |

Multivariable adjustment for age, sex, education, race, income, alcohol, smoking status, BMI, physical activity, and survey year

Supplementary Table 9. Hazard ratios and 95% confidence intervals (CIs) of all-cause mortality and cause-specific mortality among participants with chronic kidney disease vs. no chronic kidney disease, stratified by smoking status

|  |  | Total | No chronic kidney disease | | Chronic kidney disease | |  |  |
| --- | --- | --- | --- | --- | --- | --- | --- | --- |
|  | Smoking status | N  (deaths) | N  (deaths) | HR | N (deaths) | HR (95% CI) | P-value | P-value for interaction |
| All-cause mortality | Never | 3829 | 3656 | 1.00 | 173 | 2.70 (2.18-3.35) | <0.001 | 0.51 |
|  | Former | 2920 | 2764 | 1.00 | 156 | 2.98 (2.43-3.64) | <0.001 |  |
|  | Current | 2753 | 2643 | 1.00 | 110 | 2.37 (1.87-3.01) | <0.001 |  |
| Infections |  |  |  |  |  |  |  |  |
| Septicemia | Never | 45 | 39 | 1.00 | 6 | 9.30 (3.26-26.47) | <0.001 | 0.28 |
|  | Former | 52 | 48 | 1.00 | 4 | 3.60 (1.10-11.84) | 0.04 |  |
|  | Current | 32 | 29 | 1.00 | 3 | 4.38 (1.11-17.23) | 0.04 |  |
| Viral hepatitis | Never | 8 | 6 | 1.00 | 2 | 65.27 (8.53-499.48) | <0.001 | <0.001 |
|  | Former | 7 | 7 | 1.00 | 0 | - | - |  |
|  | Current | 12 | 12 | 1.00 | 0 | - | - |  |
| Human immunodeficiency virus | Never | 21 | 19 | 1.00 | 2 | 7.06 (1.17-42.65) | 0.03 | <0.001 |
|  | Former | 13 | 13 | 1.00 | 0 | - | - |  |
|  | Current | 38 | 36 | 1.00 | 2 | 2.52 (0.54-11.74) | 0.24 |  |
| Other infectious parasitic disease | Never | 13 | 11 | 1.00 | 2 | 10.25 (2.07-50.71) | <0.001 | 0.62 |
|  | Former | 8 | 6 | 1.00 | 2 | 18.03 (3.53-92.00) | <0.001 |  |
|  | Current | 9 | 8 | 1.00 | 1 | 4.81 (0.61-37.83) | 0.14 |  |
| Cancers |  |  |  |  |  |  |  |  |
| All cancers | Never | 703 | 684 | 1.00 | 19 | 1.63 (0.98-2.70) | 0.06 | <0.001 |
|  | Former | 716 | 702 | 1.00 | 14 | 1.45 (0.81-2.58) | 0.21 |  |
|  | Current | 753 | 739 | 1.00 | 14 | 1.39 (0.70-2.76) | 0.34 |  |
| Lung, trachea, bronchus | Never | 91 | 86 | 1.00 | 5 | 3.59 (1.30-9.89) | 0.01 | 0.30 |
|  | Former | 238 | 231 | 1.00 | 7 | 2.69 (1.23-5.90) | 0.01 |  |
|  | Current | 384 | 378 | 1.00 | 6 | 1.22 (0.43-3.49) | 0.71 |  |
| Kidney and renal pelvis | Never | 16 | 13 | 1.00 | 3 | 14.05 (3.83-51.53) | <0.001 | <0.001 |
|  | Former | 21 | 19 | 1.00 | 2 | 3.91 (0.81-18.86) | 0.09 |  |
|  | Current | 14 | 14 | 1.00 | 0 | - | - |  |
| Other, unspecified neoplasms | Never | 199 | 195 | 1.00 | 4 | 1.18 (0.42-3.35) | 0.76 | <0.001 |
|  | Former | 120 | 118 | 1.00 | 2 | 0.81 (0.19-3.42) | 0.77 |  |
|  | Current | 114 | 108 | 1.00 | 6 | 4.16 (1.70-10.21) | 0.002 |  |
| Endocrine, nutritional, metabolic diseases |  |  |  |  |  |  |  |  |
| Diabetes mellitus | Never | 132 | 117 | 1.00 | 15 | 7.27 (3.60-14.70) | <0.001 | 0.67 |
|  | Former | 96 | 79 | 1.00 | 17 | 8.39 (4.26-16.5) | <0.001 |  |
|  | Current | 58 | 50 | 1.00 | 8 | 8.84 (3.91-20.00) | <0.001 |  |
| Nervous system |  |  |  |  |  |  |  |  |
| Parkinson's disease | Never | 33 | 31 | 1.00 | 2 | 3.35 (0.69-16.35) | 0.13 | 0.44 |
|  | Former | 17 | 15 | 1.00 | 2 | 10.84 (2.44-48.25) | <0.001 |  |
|  | Current | 3 | 3 | 1.00 | 0 | - | - |  |
| Alzheimer's disease | Never | 75 | 73 | 1.00 | 2 | 0.78 (0.17-3.68) | 0.76 | - |
|  | Former | 53 | 53 | 1.00 | 0 | - | - |  |
|  | Current | 11 | 11 | 1.00 | 0 | - | - |  |
| Circulatory disease |  |  |  |  |  |  |  |  |
| All circulatory diseases | Never | 1283 | 1212 | 1.00 | 71 | 3.33 (2.25-4.94) | <0.001 | <0.001 |
|  | Former | 933 | 871 | 1.00 | 62 | 3.77 (2.73-5.21) | <0.001 |  |
|  | Current | 748 | 709 | 1.00 | 39 | 2.88 (1.90-4.38) | <0.001 |  |
| Primary hypertension, renal disease | Never | 44 | 39 | 1.00 | 5 | 12.67 (3.52-45.56) | <0.001 | 0.69 |
|  | Former | 27 | 21 | 1.00 | 6 | 9.68 (3.03-30.92) | <0.001 |  |
|  | Current | 13 | 10 | 1.00 | 3 | 24.80 (4.04-152.34) | <0.001 |  |
| Hypertensive heart disease | Never | 50 | 48 | 1.00 | 2 | 1.50 (0.33-6.81) | 0.60 | <0.001 |
|  | Former | 28 | 26 | 1.00 | 2 | 6.47 (1.48-28.36) | 0.01 |  |
|  | Current | 30 | 28 | 1.00 | 2 | 4.41 (0.89-21.91) | 0.07 |  |
| Hypertensive heart, renal disease | Never | 8 | 6 | 1.00 | 2 | 11.68 (1.11-122.55) | 0.04 | 0.23 |
|  | Former | 3 | 3 | 1.00 | 0 | - | - |  |
|  | Current | 2 | 1 | 1.00 | 1 | - | - |  |
| Ischaemic heart disease | Never | 633 | 592 | 1.00 | 41 | 4.06 (2.35-7.00) | <0.001 | 0.16 |
|  | Former | 539 | 505 | 1.00 | 34 | 2.97 (1.98-4.48) | <0.001 |  |
|  | Current | 441 | 421 | 1.00 | 20 | 2.09 (1.23-3.56) | 0.01 |  |
| Acute myocardial infarction | Never | 250 | 232 | 1.00 | 18 | 5.27 (2.13-13.05) | <0.001 | - |
|  | Former | 201 | 188 | 1.00 | 13 | 3.29 (1.76-6.14) | <0.001 |  |
|  | Current | 181 | 172 | 1.00 | 9 | 2.24 (1.07-4.69) | 0.03 |  |
| Atherosclerotic cardiovascular disease | Never | 93 | 90 | 1.00 | 3 | 1.24 (0.37-4.11) | 0.73 | 0.26 |
|  | Former | 72 | 69 | 1.00 | 3 | 1.70 (0.47-6.12) | 0.41 |  |
|  | Current | 81 | 78 | 1.00 | 3 | 1.78 (0.54-5.94) | 0.34 |  |
| Other chronic ischaemic heart disease | Never | 287 | 267 | 1.00 | 20 | 3.98 (2.21-7.20) | <0.001 | <0.001 |
|  | Former | 261 | 243 | 1.00 | 18 | 3.20 (1.81-5.68) | <0.001 |  |
|  | Current | 173 | 165 | 1.00 | 8 | 2.17 (0.92-5.12) | 0.08 |  |
| Heart failure | Never | 75 | 74 | 1.00 | 1 | 0.66 (0.08-5.28) | 0.69 | 0.15 |
|  | Former | 50 | 47 | 1.00 | 3 | 3.84 (1.17-12.57) | 0.03 |  |
|  | Current | 21 | 21 | 1.00 | 0 | - | - |  |
| All other forms of heart disease | Never | 190 | 184 | 1.00 | 6 | 1.38 (0.58-3.27) | 0.46 | <0.001 |
|  | Former | 110 | 103 | 1.00 | 7 | 9.68 (3.03-30.92) | <0.001 |  |
|  | Current | 80 | 77 | 1.00 | 3 | 2.21 (0.61-8.05) | 0.23 |  |
| Cerebrovascular disease | Never | 225 | 217 | 1.00 | 8 | 2.25 (0.94-5.38) | 0.07 | <0.001 |
|  | Former | 132 | 125 | 1.00 | 7 | 3.74 (1.44-9.71) | 0.01 |  |
|  | Current | 121 | 113 | 1.00 | 8 | 3.87 (1.67-8.97) | <0.001 |  |
| Other diseases of circulatory system | Never | 51 | 46 | 1.00 | 5 | 4.81 (1.59-14.58) | 0.01 | <0.001 |
|  | Former | 36 | 33 | 1.00 | 3 | 16.02 (3.73-68.89) | <0.001 |  |
|  | Current | 38 | 36 | 1.00 | 2 | 4.57 (0.92-22.64) | 0.06 |  |
| Aortic aneurysm and dissection | Never | 20 | 19 | 1.00 | 1 | 1.17 (0.15-9.33) | 0.88 | 0.80 |
|  | Former | 23 | 22 | 1.00 | 1 | 3.63 (0.46-28.95) | 0.22 |  |
|  | Current | 22 | 21 | 1.00 | 1 | 1.19 (0.15-9.49) | 0.87 |  |
| Other diseases of arteries or capillaries | Never | 17 | 16 | 1.00 | 1 | 1.17 (0.14-10.01) | 0.88 | <0.001 |
|  | Former | 10 | 8 | 1.00 | 2 | 66.93 (7.8-574.04) | <0.001 |  |
|  | Current | 15 | 15 | 1.00 | 0 | - | - |  |
| Other disorders of circulatory system | Never | 14 | 11 | 1.00 | 3 | 36.13 (8.88-147.06) | <0.001 | <0.001 |
|  | Former | 3 | 3 | 1.00 | 0 | - | - |  |
|  | Current | 1 | 0 | 1.00 | 1 | - | - |  |
| Respiratory diseases |  |  |  |  |  |  |  |  |
| Pneumonia | Never | 82 | 81 | 1.00 | 1 | 0.41 (0.06-3.07) | 0.39 | <0.001 |
|  | Former | 47 | 44 | 1.00 | 3 | 2.87 (0.73-11.23) | 0.13 |  |
|  | Current | 45 | 45 | 1.00 | 0 | - | - |  |
| Other chronic lower respiratory disease | Never | 42 | 40 | 1.00 | 2 | 3.45 (0.6-19.88) | 0.17 | <0.001 |
|  | Former | 209 | 199 | 1.00 | 10 | 2.28 (1.12-4.66) | 0.02 |  |
|  | Current | 172 | 162 | 1.00 | 10 | 3.33 (1.34-8.27) | 0.01 |  |
| Pneumonitis from solids, liquids | Never | 23 | 21 | 1.00 | 2 | 5.14 (1.01-26.11) | 0.05 | - |
|  | Former | 26 | 26 | 1.00 | 0 | - | - |  |
|  | Current | 8 | 8 | 1.00 | 0 | - | - |  |
| Other respiratory system diseases | Never | 41 | 40 | 1.00 | 1 | 0.38 (0.05-2.92) | 0.35 | <0.001 |
|  | Former | 46 | 45 | 1.00 | 1 | 1.35 (0.19-9.59) | 0.76 |  |
|  | Current | 27 | 26 | 1.00 | 1 | 1.19 (0.15-9.64) | 0.87 |  |
| Digestive diseases |  |  |  |  |  |  |  |  |
| Alcoholic liver disease | Never | 20 | 20 | 1.00 | 0 | - | - | <0.001 |
|  | Former | 14 | 12 | 1.00 | 2 | 19.05 (2.68-135.70) | <0.001 |  |
|  | Current | 46 | 43 | 1.00 | 3 | 4.44 (1.17-16.83) | 0.03 |  |
| Other chronic liver disease | Never | 24 | 23 | 1.00 | 1 | 5.51 (0.51-59.11) | 0.16 | 0.64 |
|  | Former | 27 | 26 | 1.00 | 1 | 1.57 (0.18-13.73) | 0.68 |  |
|  | Current | 34 | 30 | 1.00 | 4 | 7.69 (2.39-24.77) | <0.001 |  |
| Urinary tract disease |  |  |  |  |  |  |  |  |
| Kidney failure | Never | 71 | 56 | 1.00 | 15 | 10.88 (5.53-21.43) | <0.001 | 0.76 |
|  | Former | 56 | 43 | 1.00 | 13 | 18.12 (7.97-41.21) | <0.001 |  |
|  | Current | 27 | 22 | 1.00 | 5 | 14.76 (4.59-47.44) | <0.001 |  |
| Self-harm, interpersonal violence |  |  |  |  |  |  |  |  |
| Suicide | Never | 46 | 46 | 1.00 | 0 | - | - | <0.001 |
|  | Former | 31 | 31 | 1.00 | 0 | - | - |  |
|  | Current | 69 | 66 | 1.00 | 3 | 3.66 (1.07-12.54) | 0.04 |  |
| All other diseases (residual) | Never | 344 | 331 | 1.00 | 13 | 1.74 (0.99-3.07) | 0.05 | <0.001 |
|  | Former | 217 | 204 | 1.00 | 13 | 3.28 (1.79-6.01) | <0.001 |  |
|  | Current | 189 | 181 | 1.00 | 8 | 2.91 (1.31-6.44) | 0.01 |  |
| All other causes/all unknown causes | Never | 609 | 593 | 1.00 | 16 | 1.84 (0.95-3.58) | 0.07 | 0.20 |
|  | Former | 216 | 208 | 1.00 | 8 | 1.91 (0.83-4.39) | 0.13 |  |
|  | Current | 254 | 251 | 1.00 | 3 | 0.72 (0.21-2.44) | 0.59 |  |

Multivariable adjustment for age, sex, education, race, income, alcohol, smoking status, BMI, physical activity, and survey year

Supplementary Table 10. Hazard ratios and 95% confidence intervals (CIs) of all-cause mortality and cause-specific mortality among participants with chronic kidney disease vs. no chronic kidney disease and corresponding E-values

|  | No chronic kidney disease | Chronic kidney disease | E-values (lower CI) |
| --- | --- | --- | --- |
|  | HR | HR (95% CI) |  |
| All-cause mortality | 1.00 | 2.69 (2.38-3.04) | 4.82(4.19) |
| Infections |  |  |  |
| Septicemia | 1.00 | 5.65 (2.84-11.25) | 10.77 (5.12) |
| Viral hepatitis | 1.00 | 10.67 (2.43-46.95) | 20.83 (4.29) |
| Human immunodeficiency virus | 1.00 | 2.93 (0.94-9.14) | 5.31 (1.00) |
| Other infectious parasitic disease | 1.00 | 10.58 (3.59-31.21) | 20.65 (6.63) |
| Cancers |  |  |  |
| All cancers | 1.00 | 1.48 (1.05-2.09) | 2.32 (1.28) |
| Esophagus | 1.00 | 2.92 (0.54-15.64) | 5.29 (1.00) |
| Colon, rectum, anus | 1.00 | 0.40 (0.10-1.65) | 4.43 (1.00) |
| Liver and bile ducts | 1.00 | 1.63 (0.35-7.56) | 2.63 (1.00) |
| Pancreas | 1.00 | 0.56 (0.13-2.45) | 2.97 (1.00) |
| Lung, trachea, bronchus | 1.00 | 1.94 (1.10-3.44) | 3.30 (1.42) |
| Breast (females) | 1.00 | 2.08 (0.72-5.98) | 3.58 (1.00) |
| Ovaries (females) | 1.00 | 1.57 (0.20-12.39) | 2.52 (1.00) |
| Kidney and renal pelvis | 1.00 | 4.74 (1.81-12.41) | 8.95 (3.02) |
| Bladder | 1.00 | 3.76 (0.60-23.79) | 6.99 (1.00) |
| Brain, nervous system | 1.00 | 2.54 (0.35-18.38) | 4.51 (1.00) |
| Leukemia | 1.00 | 0.93 (0.12-7.05) | 1.34 (1.00) |
| Other, unspecified neoplasms | 1.00 | 1.76 (0.96-3.24) | 2.92 (1.00) |
| Endocrine, nutritional, metabolic diseases |  |  |  |
| Diabetes mellitus | 1.00 | 8.57 (5.60-13.11) | 16.62 (10.67) |
| Nervous system |  |  |  |
| Parkinson's disease | 1.00 | 5.01 (1.77-14.16) | 9.50 (2.95) |
| Alzheimer's disease | 1.00 | 0.40 (0.09-1.82) | 4.38 (1.00) |
| Circulatory disease |  |  |  |
| All circulatory diseases | 1.00 | 3.36 (2.70-4.18) | 6.18 (4.84) |
| Primary hypertension, renal disease | 1.00 | 13.60 (6.42-28.84) | 26.70 (12.31) |
| Hypertensive heart disease | 1.00 | 3.08 (1.13-8.38) | 5.62 (1.52) |
| Hypertensive heart, renal disease | 1.00 | 10.72 (2.47-46.49) | 20.92 (4.38) |
| Ischaemic heart disease | 1.00 | 3.15 (2.29-4.35) | 7.13 (3.86) |
| Acute myocardial infarction | 1.00 | 3.84 (2.22-6.64) | 7.13 (3.86) |
| Atherosclerotic cardiovascular disease | 1.00 | 1.53 (0.76-3.11) | 2.44 (1.00) |
| Other chronic ischaemic heart disease | 1.00 | 3.20 (2.23-4.59) | 5.85 (3.89) |
| Heart failure | 1.00 | 1.56 (0.54-4.49) | 2.50 (1.00) |
| All other forms of heart disease | 1.00 | 2.12 (1.26-3.59) | 3.67 (1.82) |
| Cerebrovascular disease | 1.00 | 3.04 (1.80-5.13) | 5.53 (3.00) |
| Atherosclerosis | 1.00 | 3.62 (0.41-31.92) | 6.69 (1.00) |
| Other diseases of circulatory system | 1.00 | 7.36 (3.22-16.81) | 14.21 (5.90) |
| Aortic aneurysm and dissection | 1.00 | 1.94 (0.51-7.31) | 3.28 (1.00) |
| Other diseases of arteries or capillaries | 1.00 | 9.89 (1.99-49.09) | 19.27 (3.40) |
| Other disorders of circulatory system | 1.00 | 41.36 (15.25-112.19) | 82.22 (29.99) |
| Respiratory diseases |  |  |  |
| Pneumonia | 1.00 | 0.90 (0.30-2.70) | 1.48 (1.00) |
| Emphysema | 1.00 | 0.61 (0.08-4.45) | 2.65 (1.00) |
| Other chronic lower respiratory disease | 1.00 | 2.72 (1.59-4.63) | 4.88 (2.57) |
| Pneumonitis from solids, liquids | 1.00 | 1.54 (0.35-6.83) | 2.46 (1.00) |
| Other respiratory system diseases | 1.00 | 1.02 (0.26-3.96) | 1.15 (1.00) |
| Digestive diseases |  |  |  |
| Alcoholic liver disease | 1.00 | 5.63 (1.90-16.66) | 10.73 (3.21) |
| Other chronic liver disease | 1.00 | 4.41 (1.74-11.17) | 8.30 (2.88) |
| Cholelithiasis, gallbladder disease | 1.00 | 2.03 (0.23-18.13) | 3.47 (1.00) |
| Urinary tract disease |  |  |  |
| Kidney failure | 1.00 | 13.07 (8.23-20.77) | 25.63(15.94) |
| Abnormal clinical, lab findings | 1.00 | 0.49 (0.07-3.56) | 3.48 (1.00) |
| Transport injuries |  |  |  |
| Motor vehicle accidents | 1.00 | 0.13 (0.02-0.91) | 15.43 (1.00) |
| Unintentional injuries | 1.00 |  |  |
| Falls | 1.00 | 2.65 (0.69-10.24) | 4.75 (1.00) |
| Other non-transport accidents combined | 1.00 | 0.69 (0.15-3.13) | 2.24 (1.00) |
| Self-harm, interpersonal violence | 1.00 |  |  |
| Suicide | 1.00 | 1.72 (0.50-5.93) | 2.83 (1.00) |
| Other causes |  |  |  |
| Other and unspecified events of  undetermined intent and their sequelae | 1.00 | 5.76 (0.57-57.99) | 11.00 (1.00) |
| Complications of medical/surgical care | 1.00 | 16.23 (1.28-205.21) | 31.96 (1.89) |
| All other diseases (residual) | 1.00 | 2.58 (1.74-3.82) | 4.60 (2.88) |
| All other causes/all unknown causes | 1.00 | 1.61 (1.05-2.47) | 2.60 (1.28) |

Multivariable adjustment for age, sex, education, race, income, alcohol, smoking status, BMI, physical activity, and survey year
